# 3D Printing of Interpenetrating Network Flexible Hydrogels with Enhancement of Adhesiveness

**DOI:** 10.1021/acsami.3c07816

**Published:** 2023-08-24

**Authors:** Lei Zhang, Huifeng Du, Xin Sun, Feng Cheng, Wenhan Lee, Jiahe Li, Guohao Dai, Nicholas Xuanlai Fang, Yongmin Liu

**Affiliations:** † Department of Mechanical & Industrial Engineering, Northeastern University, Boston, Massachusetts 02115, United States; ‡ State Key Laboratory of Primate Biomedical Research, Institute of Primate Translational Medicine, Kunming University of Science and Technology, Kunming, Yun Nan 650000, China; § Department of Mechanical Engineering, Massachusetts Institute of Technology, Cambridge, Massachusetts 02139, United States; ∥ Department of Bioengineering, Northeastern University, Boston, Massachusetts 02115, United States; ⊥ Department of Electrical and Computer Engineering, Northeastern University, Boston, Massachusetts 02115, United States; # Department of Biomedical Engineering, College of Engineering and School of Medicine, University of Michigan, Ann Arbor, Michigan 48109, United States

**Keywords:** 3D printing, interpenetrating network, polydopamine, flexible hydrogel, adhesive hydrogel, biocompatibility

## Abstract

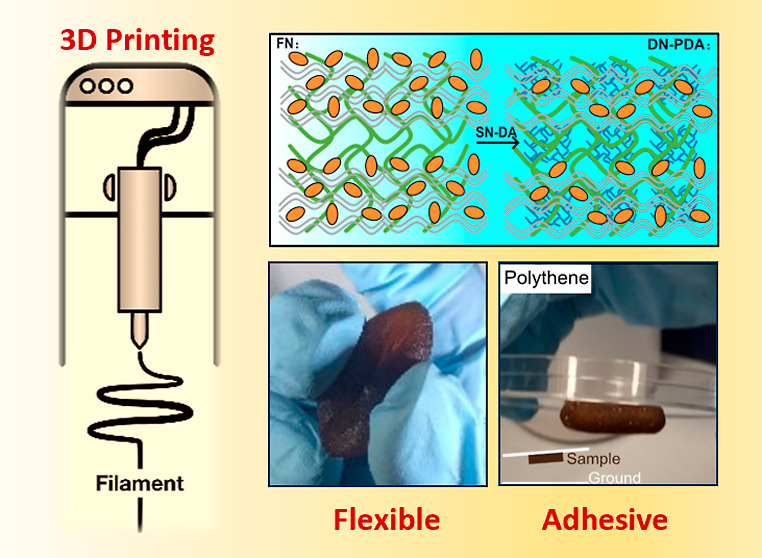

3D printing of hydrogels has been widely explored for the rapid fabrication of complex soft structures and devices. However, using 3D printing to customize hydrogels with both adequate adhesiveness and toughness remains a fundamental challenge. Here, we demonstrate mussel-inspired (polydopamine) PDA hydrogel through the incorporation of a classical double network (2-acrylamido-2-methylpropanesulfonic acid) PAMPS/(polyacrylamide) PAAm to achieve simultaneously tailored adhesiveness, toughness, and biocompatibility and validate the 3D printability of such a hydrogel into customized architectures. The strategy of combining PDA with PAMPS/PAAm hydrogels leads to favorable adhesion on either hydrophilic or hydrophobic surfaces. The hydrogel also shows excellent flexibility, which is attributed to the reversible cross-linking of PDA and PAMPS, together with the long-chain PAAm cross-linking network. Among them, the reversible cross-linking of PDA and PAMPS is capable of dissipating mechanical energy under deformation. Meanwhile, the long-chain PAAm network contributes to maintaining a high deformation capability. We establish a theoretical framework to quantify the contribution of the interpenetrating networks to the overall toughness of the hydrogel, which also provides guidance for the rational design of materials with the desired properties. Our work manifests a new paradigm of printing adhesive, tough, and biocompatible interpenetrating network hydrogels to meet the requirements of broad potential applications in biomedical engineering, soft robotics, and intelligent and superabsorbent devices.

## Introduction

Hydrogels have emerged as excellent candidates for soft tissue engineering scaffolds, drug delivery systems, molecular filters, and superabsorbent devices for their outstanding properties, such as water absorption and retention ability, biocompatibility, high resemblance with the native extracellular matrix, and tunability.^1^ Generally, the application of hydrogels in load-bearing situations is often hindered because hydrogels normally lack the desired strength, toughness, and recoverability.^2–4^ However, this situation has dramatically changed since the invention of the double network (DN) hydrogel,^5^ which has stimulated extensive research on the toughening mechanisms of hydrogels.^6–8^ For instance, Gong and co-workers proposed a two-step sequential free-radical polymerization method of poly(2-acrylamido-2-methylpropanesulfonic acid) (PAMPS) and polyacrylamide (PAAm) to achieve unusual mechanical properties.^9^ PAMPS, a rigid network, swells nearly to the breaking point, while PAAm, a ductile network, is highly stretchable. As a result, even with high water content, the PAMPS/PAAm DN hydrogels have outstanding stiffness, strength, and toughness, comparable to those of cartilage and rubber.^10^ Furthermore, the mechanical properties can be changed by varying the ratio of the monomers of the two networks and the densities of their cross-linkers.^10–13^

Despite the recent advancement in the development of new types of hydrogels, 3D printing tough hydrogel-based complex structures remains a significant challenge. The reasons lie in the inherent robust mechanical features of hydrogel inks, which make the inks difficult to extrude.^14–16^ In addition, most mixed Laponite nanoceramic hydrogels lack bioactive and functional sites to interact with living tissues.^4^ Cumbersome postprocessing, such as collagen coating, is necessary for biomedical applications. Accordingly, combining biomimetic cues with synthetic hydrogels to support physiologically relevant interactions between cells and materials is a good strategy.^6,11,17,18^ Inspired by the strong attachment protein in mussel adhesive pads, people have used polydopamine (PDA) to effectively improve hydrophilicity, cell adhesion and differentiation, protein recognition, and adsorbability.^19^ For example, some researchers have combined biomimetic PDA with covalently cross-linked hydrogels like PEG and PAAm to achieve both tough and tissue-adhesive characteristics, but only in simple structures such as cylinders, cuboids, and planar ones.^17,18,20–28^

Fabricating complex 3D architectures is greatly facilitated by the precise deposition of hydrogels and bioactive inks through an automated layer-by-layer process.^29,30^ Printing tough hydrogels into complex architectures typically requires UV/thermal curing or sacrificial/support materials.^31–34^ Herein, we demonstrate the unique doping of mussel-inspired PDA nanocomposites that can be integrated into the 3D printing of an interpenetrating network hydrogel based on PAMPS/PAAm. Such doping into 3D printing of PAMPS/PAAm leads to simultaneously tailored mechanical robustness and biomimetic adhesion without extra curing/supporting methods. To meet these objectives, two relevant issues must be considered.

First, the 3D bioprinting in our study makes use of the computer-controlled extrusion method for layer-by-layer deposition of hydrogels, in which inks need to flow as a liquid during extrusion and then solidify after extrusion. In order to meet the demand of 3D direct printing, the ink is usually extruded onto/into a second support/sacrificial material through a double printing head. By using a curing method such as UV irradiation and temperature change, the printed hydrogel structure can be obtained upon removal of the support/sacrificial material. Alternatively, the curing method can be used to rapidly solidify the hydrogel ink during printing. In this circumstance, the fidelity and fluency of the 3D printing process are limited by the curing speed. Moreover, the ink close to the tip of the nozzle tends to gel, which clogs the nozzle. To solve this problem, we mixed Laponite nanoceramic with a 1 wt % alginate aqueous solution and used the combination as the rapid rheology modifier. Laponite is a nanosilicate commonly applied as a rheology modifier for waterborne products. Alginate is a natural polysaccharide; specifically, it is an amorphous copolymer with a linear, unbranched chain composed of mannuronic (M) and guluronic (G) acids. Laponite’s dual ionic characteristics can interact with M and G acids in alginate aqueous solutions. By taking advantage of the electrostatic interactions between Laponite and alginate chains, we can rapidly establish a relatively stable rheology of the hydrogel ink to ensure continuous extrusion and superior 3D printability without extra curing or support/sacrificial materials and consequently print complex structures.^35,36^ In addition, the rheology modifier is not only embedded into the ink matrix to obtain ideal 3D printability but also triggers a homogeneous PDA to form strengthened DN–PDA composite 3D architectures.

Second, a strong bonding ability of biomimetic PDA to proteins, cells, tissues, and polymers has been demonstrated (e.g., PAAm, Alginate, and PEG).^19–24^ Hence, during the polymerization process of PDA, the printed architecture precursor can readily bond with PDA and form PAAm-PDA hydrogels.^25–28,37^ Unfortunately, this may cause damage to the 3D DN–PDA architectures (Figure S1, Supporting Information). Therefore, to simultaneously integrate adhesive characteristics and toughness into the customized architectures, it is critical to balance the interpenetrating network formation and polymerization of PDA without sacrificing the printed architecture. The proposed approach of the 3D DN–PDA hydrogel and its direct printing could be generalized to produce a wide integration of multifunctions and customized architectures.

## Results and Discussion

**Figure 1:**
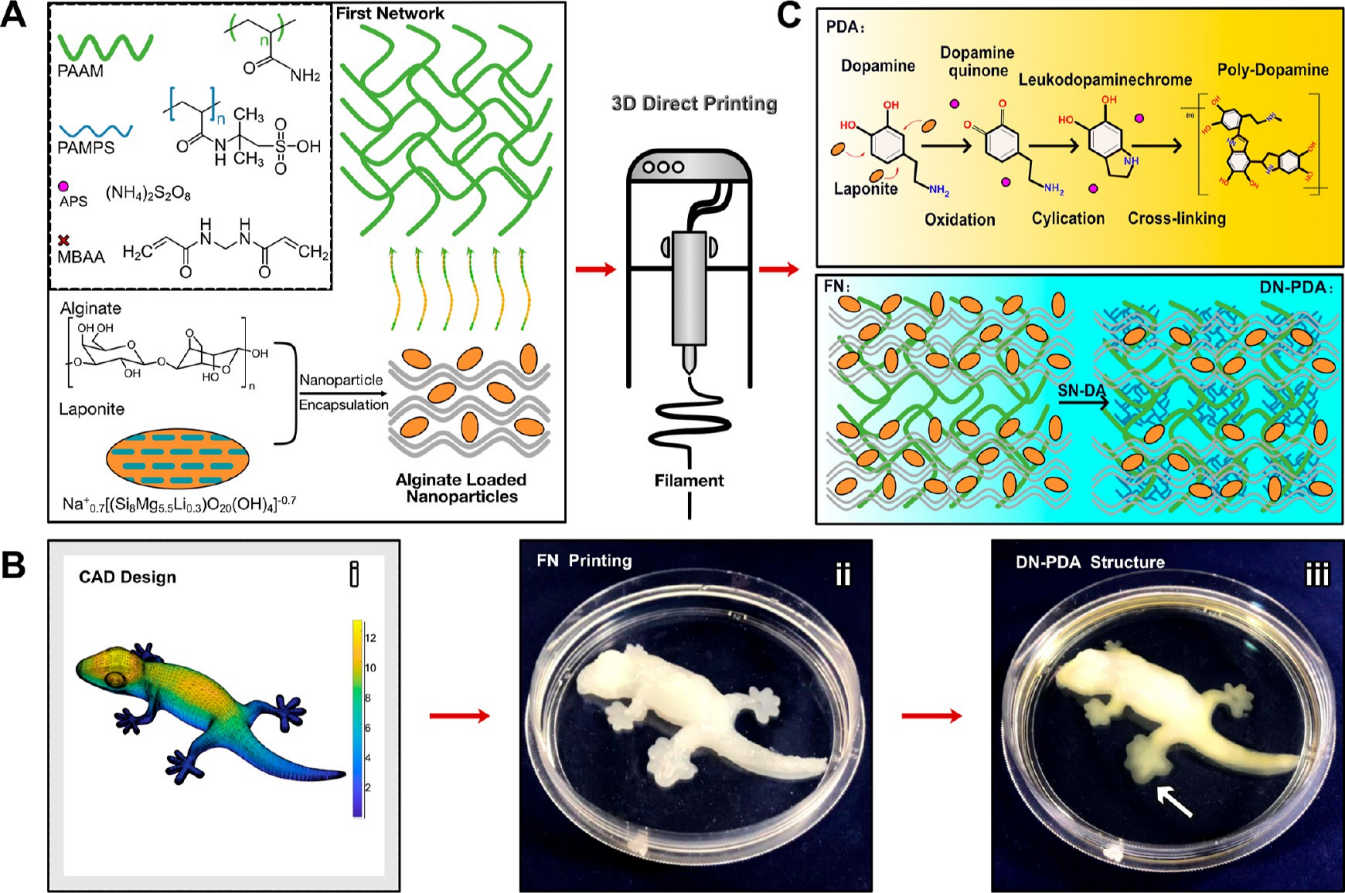
Synthesis and 3D printing of DN (PAMPS/PAAm)-PDA hydrogel. (A) Illustration of the FN ink synthesis and cross-linking of DN with PDA. (B) 3D PAMPS/PAAm-PDA hydrogel architecture fabrication: from CAD model (i) to FN printing (ii) and to DN–PDA cross-linking (iii). The whole 3D printing process does not need extra curing techniques or sacrificial/support materials. (C) Schematic of the synthesis mechanism of the DN (PAMPS/PAAm)-PDA hydrogel.

We demonstrate a DN(PAMPS/PAAm)-PDA hydrogel that combines desired toughness, flexibility, adhesiveness, and bioactivity and successfully turns the hydrogel into customized 3D architectures. Figure 1 shows the synthesis process and printability of the hydrogel ink. The detailed experimental procedures can be found in Section S1 of the Supporting Information. Briefly, we dissolved powders of acrylamide, *N*,*N*-methylenebis(acrylamide) (MBAA), and ammonium persulfate (APS) in deionized water. In order to directly print 3D architectures without an extra curing process, we mixed Laponite nanoceramic with a 1 wt % alginate aqueous solution and used the combination as the rapid rheology modifier (Figure 1A). The rheology modifier was added to the acrylamide mixtures and kept stirring for 20 min at 600 rpm to form the first network (FN) ink. Laponite’s dual ionic characteristics can interact with M and G acids in alginate aqueous solution to establish a stable viscosity value to ensure continuous extrusion.^30,33,35^

The FN ink can be rapidly 3D-printed into scalable, complex, customized architectures, such as lizards [Figure 1B(i)], at a linear 3D-printing speed of 10 mm/s with a relatively low extrusion pressure of 10 psi and individual filamentary diameters of 260 μm. We have also printed fish, human ear, and square grid planar, as demonstrated later in the paper. After printing [Figure 1B(ii)], the 3D architectures were directly soaked into the second network (SN)-dopamine solvent, which contained dopamine, 2-acrylamido-2-methylpropanesulfonic acid (AMPS), *N*,*N*,*N*′,*N*′-tetramethylethylenediamine (TEMED), MBAA, APS, and deionized water. Laponite nanoceramic was encapsulated in the 3D printed FN architectures, where its oxidation was limited in the confined space, resulting in controllable polymerization within the 3D printed structure. When immersed in the SN-dopamine solvent, two independent networks formed simultaneously and resulted in a balance while competing cross-linking chemistries were used. SN solvent penetrated from the edge to the center of the 3D architectures and then polymerized the SN within the pre-existing hydrogel network. The 3D printed architecture changed from a ceramic-like white color to translucency, as shown in the lizard’s toe pointed out by the white arrow in Figure 1B(iii). Simultaneously, dopamine monomer diffused into the concentration area of the Laponite and produced oxidative polymerization within the alkaline 3D architectures. During the polymerization process, dopamine was first oxidized to dopamine quinone under the action of oxygen. Then intramolecular cyclization occurred due to the 1, 4-Michael addition reaction, and dihydroxyindole (DHI) was generated through oxidation and intramolecular rearrangement reactions. Following immersion in the SN-dopamine solvent, the solution underwent a transformation from a colorless state to an initial orange-red hue, which then gradually changed to a deep red color. At the same time, the 3D-printed product changed from a ceramic-like white to color translucency and ultimately to a brownish black appearance (DN–PDA). All these transitions result from the quinone compounds formed by the oxidation of dopamine. This phenomenon indicates that the APS and Laponite ceramics could catalyze the oxidation of dopamine under acidic conditions because the oxidative polymerization reaction of dopamine is generally considered to be a free radical process.

As illustrated in Figure 1C, two independent networks (DN and PDA) are formed simultaneously and competitively consume the strong oxidizing agent APS during the DN–PDA synthesis. Therefore, the harmony between PDA formation and DN formation is pivotal to forming DN–PDA composite 3D architectures with integrated flexible and adhesive characteristics. With the optimal composition adjustment, the different cross-linking times of DN and PDA lead to different appearance performances of the 3D printed architectures (Figure S2, Supporting Information). The ultralong cross-linking process of the 3D DN–PDA architecture gives rise to local swelling, although all the printed architectures still maintain flexible and reversible mechanical features. To further verify the effect of SN-DA cross-linking, we fabricated three groups of FN hydrogel samples with a diameter of 34 mm and a thickness of 9 mm, immersed them in the second network solvent (SN-DA), and observed the stability of DN–PDA hydrogel with different immersion times (Figure S3, Supporting Information). It was found that the color change and curing appearance are the same as those of the experiment we conducted before. Upon immersion in the SN-dopamine solvent, the solution changed from a colorless state to orange-red and gradually drew to a deep red. The three samples were taken out of the solvent after 5 h, and the diffusion extent appeared to be uniform throughout the samples when the volume of the samples was the same. In addition, we fabricated FN samples with a diameter of 34 mm and a thickness of 3, 6, and 9 mm, respectively, and studied the effect of soaking time on the samples with different volumes (Figure S4, Supporting Information). The samples were cut into halves to observe the cross-linking and diffusion extent inside the samples. It was found that the curing time of polymerization (DN–PDA) of the hydrogel was prolonged with an increase of thickness or volume. For large-volume samples, the curing time can be extended so that large-volume samples can cure completely. Similarly, the long cross-linking process of FN hydrogels still maintains adhesive, flexible, and reversible mechanical features (see Movie S1).

**Figure 2:**
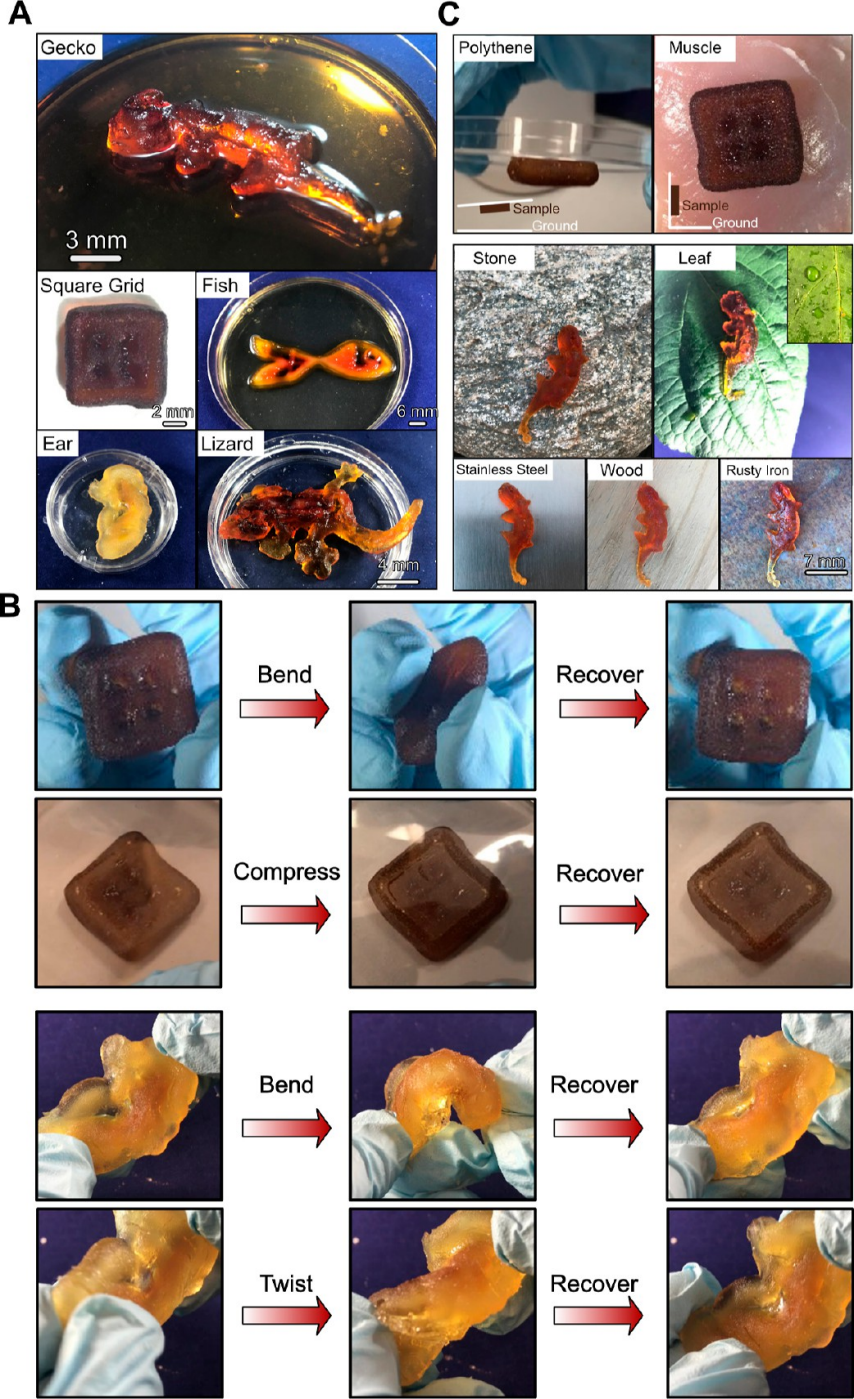
Deformation ability and adhesive characteristics of 3D PAMPS/PAAm-PDA hydrogel architectures. (A) After the anchoring and confining of dopamine and polymerization of SN molecules in the printed FN architectures, the integrated 3D PAMPS/PAAm-PDA hydrogel architectures such as gecko, fish, square grid sample, and human ear are obtained. (B) 3D PAMPS/PAAm-PDA hydrogel architectures showing good structural integration when they are deformed. (C) Printed hydrogel architectures exhibiting adhesive characteristics to hydrophobic and hydrophilic surfaces, such as hydrophobic polythene and leaves, as well as hydrophilic tissue (chicken’s fresh muscle tissue), metal, rock, and wood. The inset in the image shows the hydrophobicity of leaves.

**Figure 3:**
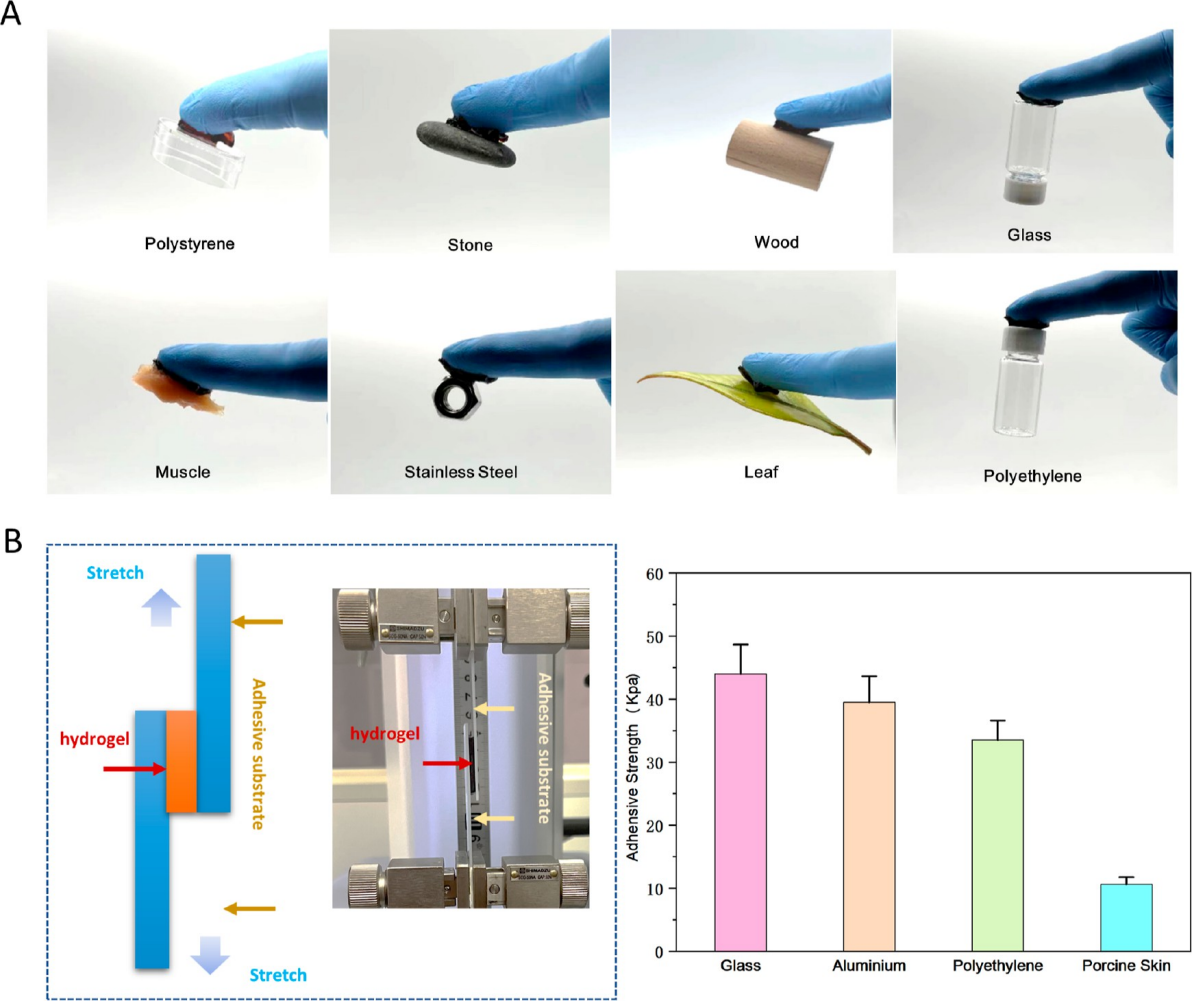
Adhesive characteristics of the PAMPS/PAAm-PDA hydrogel. (A) Adhesion of the PAMPS/PAAm-PDA hydrogel to different substrates, including polystyrene, stone, wood, glass, stainless steel, leaf, aluminum, porcine skin, and muscle tissues. (B) Adhesion strength of the PAMPS/PAAm-PDA hydrogel quantified by a tensile mechanical testing machine. The left panel shows the measurement setup, and the right figure presents the highest adhesive strength of the PAMPS/PAAm-PDA hydrogel to glass, aluminum plate, polyethylene, and porcine skin.

PDA contains amino groups and catechol groups.^37,38^ The amino groups can form balanced electrostatic interactions with the Laponite nanosheets. The catechol groups are able to chelate with the degradation ions, such as Mg and Si, of Laponite through the adjacent hydroxyl groups.^39^ The PDA chains can be linked to the DN through the interactions between the catechol group of the PDA chains and the amino and hydrogen groups from the DN (PAAM/PAMPS network). The above reactions facilitate the anchoring and confining of dopamine and SN molecules polymerizing in the printed FN architectures, which lead to the integrated DN–PDA hydrogel 3D architectures, such as gecko, fish, square grid, lizard, and human ear, as shown in Figure 2A. The printed hydrogel architectures are highly deformable and flexible, demonstrating that the synthesized PDA nanocomposites together with the Laponite ceramic addition do not significantly affect the superior mechanical properties of the DN hydrogel.^5^ As shown in Figure 2B and Movie S2, the printed hydrogel architectures can be repeatedly bent, compressed, and twisted to a large extent multiple times and held for 30 s at each deformation. After the applied force was removed, the hydrogels recovered automatically and rapidly to their original states. Endowing adhesive characteristics with excellent mechanical properties has been challenging for most of the published works.^16–18^ In addition to having excellent flexible mechanical behaviors, the printed DN–PDA hydrogel architectures also exhibit desirable adhesive characteristics to a variety of surfaces, including hydrophobic polythene and leaves as well as hydrophilic rock, metal, and wood, as demonstrated in Figure 2C and Movie S3. More interestingly, the printed hydrogel architectures are able to adhere to organ tissues. Some additional measurements on the adhesion properties can be found in Figure 3. In particular, we quantified the adhesion strengths with a tensile mechanical testing machine. The highest adhesive strength of the DN–PDA hydrogel to glass, aluminum plate, polyethylene, and porcine skin are 43.97 ± 4.67, 39.49 ± 4.14, 33.52 ± 3.08, and 10.62 ± 1.14 kPa, respectively. The adhesion of the PAMPS/PAAm-PDA hydrogel is attributed to the presence of a sufficient number of free-catechol groups via multiple hydrogen-bonding interactions, ion–dipole interactions, metal complexation, and van der Waals interactions with the adherents, which are produced during the DA oxidation process induced by APS and laponite clay nanosheets confined in the FN.^19–24^

**Figure 4:**
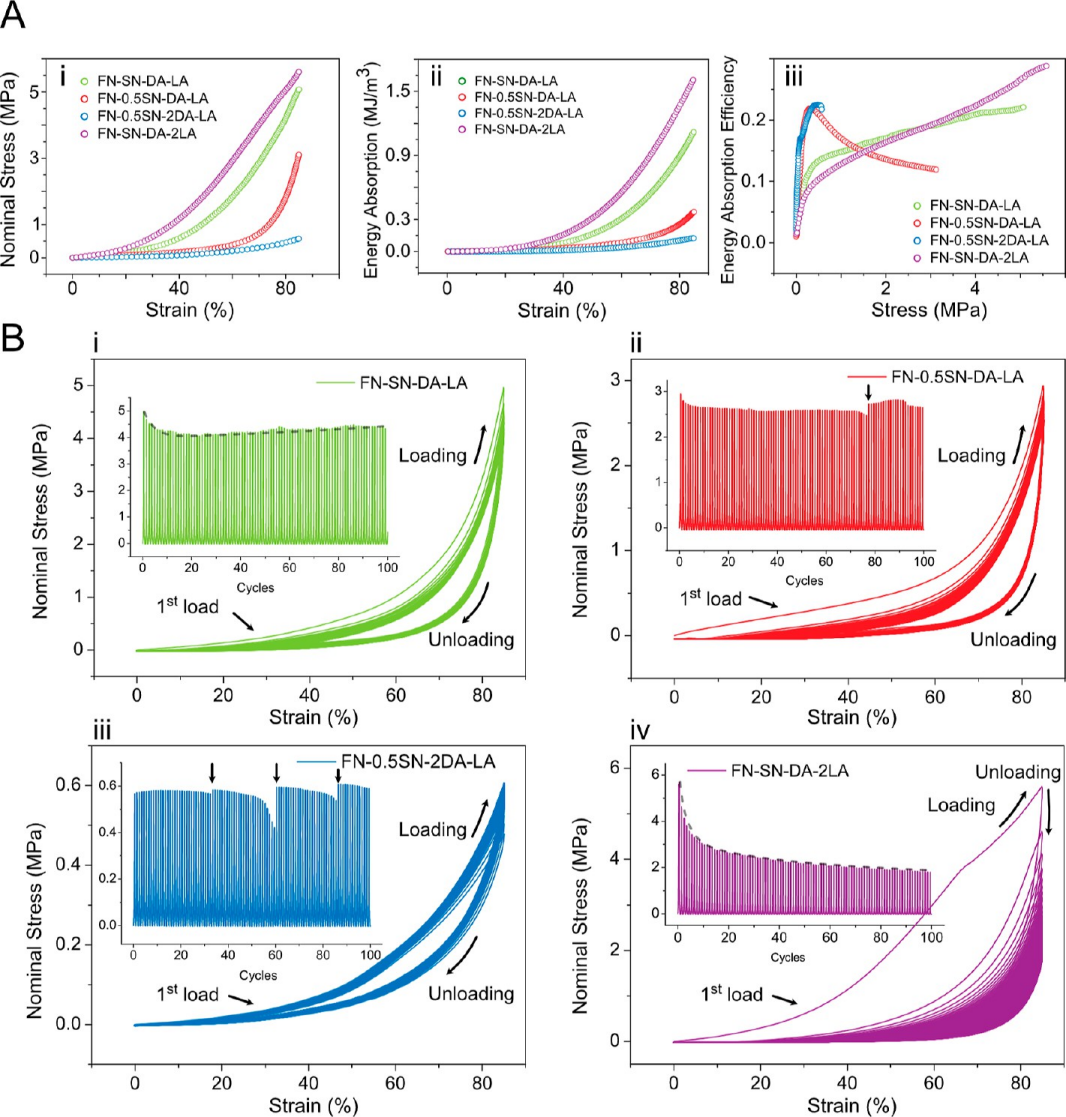
Mechanical properties of PAMPS/PAAm-PDA hydrogels. (A) Nominal stress–strain curves of the FN-SN-DA-LA hydrogel, FN-0.5SN-DA-LA hydrogel, FN-0.5SN-2DA-LA hydrogel, and FN-SN-DA-2LA hydrogel when compressed to 85% (i). The energy absorption per volume (ii) and the energy absorption efficiency (iii) were deduced from the nominal stress–strain curves. (B) 100 cyclic loading–unloading compressive stress–strain curves of the FN-SN-DA-LA hydrogel (i), FN-0.5SN-DA-LA hydrogel (ii), FN-0.5SN-2DA-LA hydrogel (iii), and FN-SN-DA-2LA hydrogel (iv). Each cycle was loaded up to 85% strain without stopping. The insets show the nominal stress during loading–unloading behavior corresponding to each cycle.

The mechanical responses of the DN(PAMPS/PAAm)-PDA hydrogel were assessed by performing a uniaxial compression assay, as displayed in Figure 4. We hypothesize that the mechanical performance of this hydrogel relies on a combination of three mechanisms: The covalent cross-linking and reversible network in DN maintain elasticity and strength under large deformations. Meanwhile, the reversible PDA cross-linking with DN further facilitates the dissipation of mechanical energy. Additionally, the added Laponite–Alginate (LA) component contributes to the nanoparticle enhancement. The anisotropic and disk-like, high aspect-ratio morphology of the Laponite nanoparticles gives rise to the high surface interactions between the polymers and the nanoparticles. Experimentally, for validation of the hypothesis of the interpenetrating hydrogel with various constituting networks, we varied the content ratio of SN, DA, and LA in the hydrogel and used four groups of FN-SN-DA-LA, FN-0.5SN-DA-LA, FN-0.5SN-2DA-LA, and FN-SN-DA-2LA hydrogels to assess the mechanical response. Figure 4A(i) plots the nominal compressive stress–strain responses of the tested hydrogels. In the stress–strain curves of the single loading to 85% strain, the hydrogels show smooth deforming behaviors with a long deformation plateau up to ∼60% strain. After the plateau regime, the curves enter the densification regime, where stress increases significantly under a small strain change. In the case of the FN-SN-DA-LA group and the FN-SN-DA-2LA group, the plateau regimes behave differently from the FN-0.5SN-DA-LA and FN-0.5SN-2DA-LA groups. A slight continuous increase of stress took place with the increase in strain, resulting in a large compressibility and high strength. The energy absorption per volume (*W*) can be calculated by integrating the nominal stress with respect to strain of stress–strain curves up to densification, that is, .

In Figure 4A(ii), the energy absorption as a function of strain is plotted for the tested hydrogels. Under the first loading of 85% strain, the energy absorption curves show a consistent trend with the stress–strain responses. The softer hydrogels, that is, the FN-0.5SN-DA-LA and FN-0.5SN-2DA-LA groups, are able to absorb energy with large deformations because there is a long plateau that extends up to densification. In contrast, the FN-SN-DA-LA and FN-SN-DA-2LA groups show a strengthening-like behavior, which absorbs energy with relatively low deformation and high stresses.

We also studied the energy absorption efficiency (*E*), which is defined as the ratio of the absorbed energy up to the stress divided by the stress itself (*W*/σ). The efficiency of the hydrogels as a function of the stress is plotted in Figure 4A(iii). The maximum of the efficiency identifies the condition for optimal energy absorption of the hydrogels when the maximum stress reaches a limited value. For the FN-0.5SN-DA-LA and FN-0.5SN-2DA-LA groups, the efficiency shows a maximum at a certain stress (0.32 and 0.48 MPa), corresponding to the densification strain around 56 and 81% strain, respectively. Beyond the stress level, the increase in absorbed energy is lower than the stress increase. Regarding the FN-SN-DA-LA and FN-SN-DA-2LA group, the efficiency shows the first peak at a similar stress range (<0.5 MPa), corresponding to the lower level of deformation (around 37 and 18% strain, respectively). Beyond the stress level, the absorbed energy efficiency slightly increases until the sample is compressed to a limited strain (85%).

The stress–strain response in Figure 4A was conducted under single-loading up to a certain strain level. However, it is highly desirable to have hydrogels with the ability to recover back to their original state after the release of the strain. To further test the mechanical responses, we loaded the hydrogels to 85% strain for 100 continuous cycles and recorded the evolution of stress–strain curves over cycles (Figure 4B). The stresses during the loading–unloading process over the 100 cycles are shown as insets in Figure 4B. For the FN-SN-DA-LA group [Figure 4B(i)], despite slight mechanical softening induced by viscoelasticity during the first several cycles (e.g., first to sixth cycles), the stress–strain loading–unloading curves in the subsequent cycles are nearly identical, indicating a relatively steady state. Such a steady state is also reflected in the maximum stress reached in each cycle [the inset in Figure 4B(i)], indicating an effective energy dissipation mechanism. The cyclic softening is due to the incomplete reforming of noncovalent interactions within the short time scale of one loading cycle-. Compared to the first cycle, we can readily recognize that the hydrogels were able to recover to their original state over 100 cycles. When we decreased the content ratio of SN (0.5SN), less covalent bonding of DN formed. The maximum strength of the FN-0.5SN-DA-LA group decreased from 5 to 3 MPa compared to that of the FN-SN-DA-LA group when the samples were loaded to 85% strain. Nevertheless, it was maintained for more than 70 cycles. Stress relaxation, as indicated by the black arrow in Figure 4B(ii), was observed when the samples were loaded for 80 cycles. The hydrogel relieved the state of stress under constant strain. When we kept the decreased SN (0.5SN) and doubled the content of DA (2DA), FN-0.5SN-2DA-LA, less covalent PAAm bonding formed in DN, while more single reversible chains between PDA and polymers bonded. Besides, because both DN and PDA polymerizations consumed the strong oxidizing agent, fewer Laponite ceramic particles remained in the DN-PDA hydrogels. As we can see from Figure 4B(iii), even though more stress relaxation responses happened during the cyclic loading–unloading process, the maximum stress recovered to its original state after the relaxation, which indicates that the DN–PDA hydrogels exhibit self-recovery and excellent superelasticity. Compared with the optimal composition of FN-SN-DA-LA, we doubled the content of LA to obtain a significant nanoparticle enhancement mechanism. More residual Laponite nanoceramic particles remained in the hydrogels after stimulating the polymerization of PDA. For the first loading–unloading cycle of FN-SN-DA-2LA [Figure 4B(iv)], the large area of stress–strain curves up to the densification indicates a high quantity of energy absorption per unit volume, which is consistent with the analysis in Figure 4A. However, with the increasing loading–unloading cycles, the hydrogels showed significant fatigue behavior. As the loading–unloading increased to 40 cycles, the maximum stress became steady but could not recover from the initial stress. The maximum stress tends to be stable, and the cyclic stress–strain curves exhibit a dense state compared to that of the rest of the three hydrogels [Figure 4B(i–iii)]. It is because the FN-SN-DA-2LA hydrogel has been compressed to a densification regime that a large amount of residual strain is generated.

**Figure 5:**
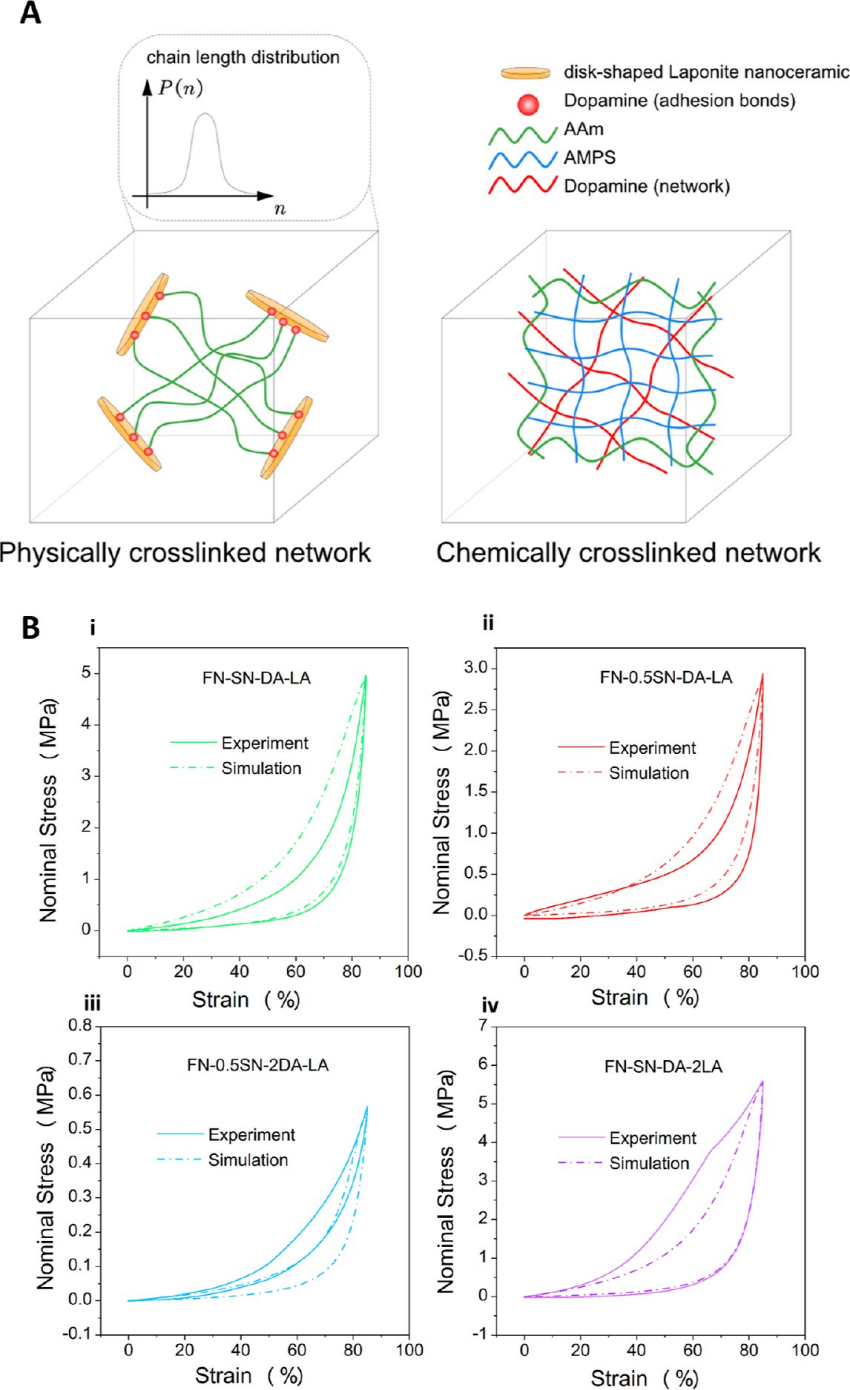
Modeling of the large deformation of the interpenetrating network with different cross-linking mechanisms. (A) Schematics of the interpenetrating hydrogel with different component polymer networks. Two essential cross-linking mechanisms exist in this theory: one with the nanoparticle cross-linker (physically cross-linked network) and the other with interpenetrating networks (chemically cross-linked network). (B) Nominal stress–strain curve during the first cycle of loading/unloading (max strain 85%) of materials with different formulations.

To account for the physical mechanisms involving the interaction between the interpenetrating polymer networks and the disk-shaped nanoparticles, we propose the following theory for the stretch/compression of interpenetrating hydrogel networks with both physical and chemical cross-linkers (Figure 5A). In this work, we aim to provide a quantitative description of the mechanical behavior of interpenetrating networks with intercalated nanoparticles. The free energy function of DN–PDA nanocomposite hydrogels can be modeled as
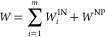
1where superscript IN/NP stands for the free energy due to stretching/compression of the interpenetrating networks (IN) and nanoparticle cross-linker (NP), respectively. The model considers the polymerization of dopamine that penetrates the PAAm–PAMPS double network to form a complex interpenetrated polymer network.

The stress–strain relationship in the loading direction is then given by . To facilitate a quantitative comparison with experimental results, the first Piola–Kirchhoff stress contribution from PAAm–PAMPS-PDA can be expressed as the following formula in the case of uniaxial compression (0 < λ ≤ 1), with the aid of the eight-chain network model^40–45^
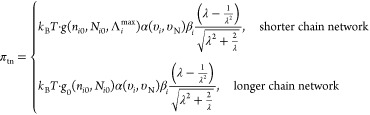
2where  is the inverse Langevin function of the microstretch (a function of the compression ratio in the experiment, which will be shown later) of a polymer chain. The network alteration function *g*(*n*_*i*0_, *N*_*i*0_, Λ_*i*_^max^) defines the damage and rearrangement of shorter chains. It depends on the initial chain parameters (*n*_*i*0_, *N*_*i*0_) and the maximum stretch during the whole loading history starting from the original state, which is assumed to be constant for a longer chain network. The amplification factor  is caused by the swelling of the hydrogel (as indicated by the volume fraction of the particular *i*-th network υ_*i*_), as well as the reinforcement of the rigid nanoceramic filler. Collectively, the amplification of the stretch of the polymer chain can be related to the macro-stretch (an experimentally obtainable quantity) via the relationship: , with .^36–38^

On the other hand, the contribution from the nanoceramic-cross-linked network can be written as^37,39^
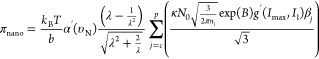
3where , *V*_0_ is the volume taken up by an individual particle, and υ_N_ is the volume fraction of the nanoceramic cross-linker. More details of the theory can be found in Section S7 of the Supporting Information. Based on this model of different cross-linking mechanisms, we performed numerical simulations of the nominal stress–strain relationship under the first cycle of compressive loading/unloading of the material. The results were plotted on the same graph along with data from experimental measurements of the nominal stress–strain curves during uniaxial compression of a hydrogel sample. As shown in Figure 5B, four different formulations were adopted as bases for comparison. We adjusted the volume fractions of different components in accordance with the experimental formulations to fit the maximum stress values attained in each test. The theory is able to predict the unloading curves pretty well except for (iii). It slightly overestimates the hysteresis in parts (i and ii), whereas it underestimates the hysteresis loop in (iv), possibly due to the addition of large volumes of Laponite ceramics, which further complicates the estimation of the network alteration function and results in complex rheological properties of the material. The peak stress shows a drastic decline in (iii) when dopamine is doubled. We hypothesize that the competition of the oxidizing agent APS between dopamine and the covalently cross-linked network leads to the depletion of a shorter chain network that otherwise exists and contributes to the stiffness of the material greatly. Further investigation is needed to establish a comprehensive model capable of predicting the hysteresis loop more accurately and the long-term behavior of the hydrogel under cyclic loading. Additional experimental observations and discussions along this direction can be found in Sections S7 and S8 of the Supporting Information and Figure S5.

To better understand the mechanical performance mechanism, we have used scanning electron microscopy (SEM) together with energy-dispersive X-ray spectroscopy (EDS) to characterize the microstructure of the hydrogels. The results are presented in Figure S6 in the Supporting Information. When excess LA is doped in the hydrogel, like FN-SN-DA-2LA, more residual Laponite nanoceramic particles remain in the hydrogels after stimulating the polymerization of PDA. It induces a small amount of C peak (quantitatively 17 at. %) while a relatively large amount of Si (17 at. %) and Mg (13 at. %) ceramic elements remain (Figure S6A). Macroscopically, the hydrogel maintains structural integrity after being repeatedly compressed. Microscopically, however, ceramic particles clump together and form discontinuous clay chunks. Many vertical cracks occur, and substantial debris is created. On the contrary, when less Laponite remains in the hydrogel, typically FN-0.5SN-2DA-LA, C element (49 at. %) is dominant in the polymer, while little ceramic elements, such as Mg, Na, and Si (<5 at. %), are detected (Figure S6B). Microscopically, from the magnified SEM images, the surface of the hydrogel appears relatively flat and smooth. Macroscopically, the hydrogel exhibits very soft and reversible characteristics when compressed cyclically.

**Figure 6:**
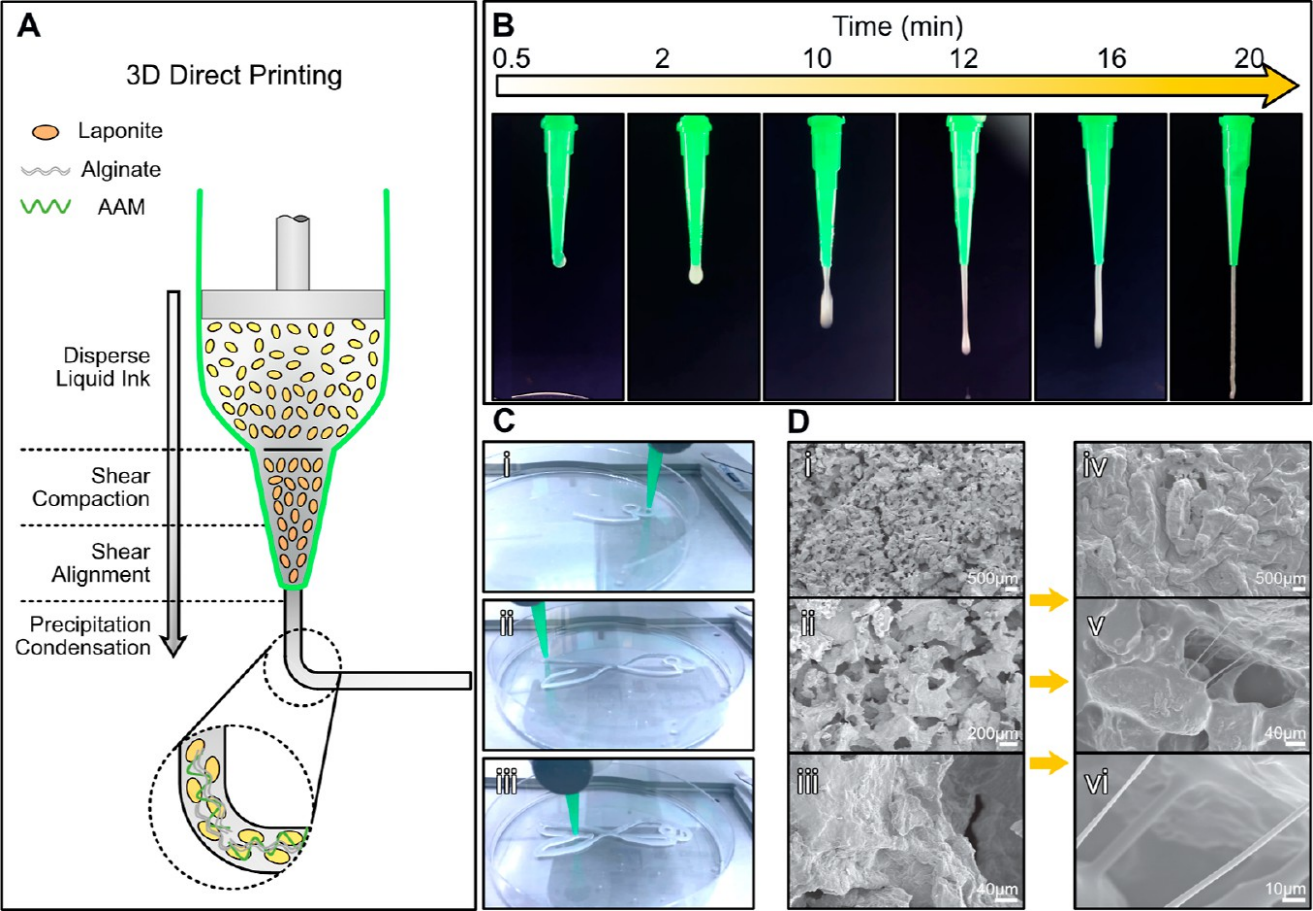
Process and microstructure from printing ink to PAMPS/PAAm-PDA hydrogel. (A) When the ink flows through the nozzle tip, shear force promotes Laponite flakes, together with macromolecular chains (alginate and AAM), reorientation and alignment of the flakes along the direction of flow. (B) Photograph series showing the evolution of ideal extrusion filament after stirring at 600 rpm for 0.5, 2, 10, 12, 16, and 20 min. (C) Direct 3D printing of a fish model without UV curing (nozzle diameter: 22 G; extrusion pressure: 10 psi; extrusion speed: 10 mm/s). (D) SEM images showing the microstructure before (i–iii) and after (iv–vi) DN–PDA cross-linking. Microfibril bundles, ranging from 1 to 10 μm in diameter, are observed in the interpenetrating channel from the neighboring PAMPS/PAAm-PDA polymer matrix.

In PAMPS/PAAm-PDA hydrogels, regardless of the PAMPS/PAAm network or dopamine polymerization (PDA), they both need to be fully dissolved in an aqueous solution and cross-linked with an oxidizing agent. In this case, the problem of either low viscosity or difficultly controlled chemical cross-linking hampers the formation of filaments and the deposition of the material. Laponite is a nanosilicate commonly used as a rheology modifier of waterborne products. Sodium alginate is an amorphous copolymer with a linear, unbranched chain composed of M and G acids. Without loss of generality, we use the combination of Laponite and alginate as the innumerable “micro-bridge”, homogeneously fusing with the macromolecular chain, to allow for extrusion-based printing to produce self-supporting structures without UV curing at room temperature (Figure 6A–C). To simplify the experiment process, we fixed the concentration of the alginate solution and gradually doped the ink with Laponite. When the disk-like Laponite was dispersed in the solution, randomly oriented particles suspended in the ink. After stirring at 600 rpm for about 20 min, the ideal filament with a diameter nearly identical to the inner diameter of the conical nozzle was continuously extruded at a low extrusion pressure (as low as 10 psi). Importantly, the interactions generated between the anionic alginate and the charged surfaces of the Laponite hindered the premature covalent cross-linking of FN, which ensured the fluency of the printing process. When the ink flowed through the nozzle tip, the shear force promoted Laponite flakes together with macromolecular chain reorientation and alignment of the flakes along the direction of flow.

**Figure 7:**
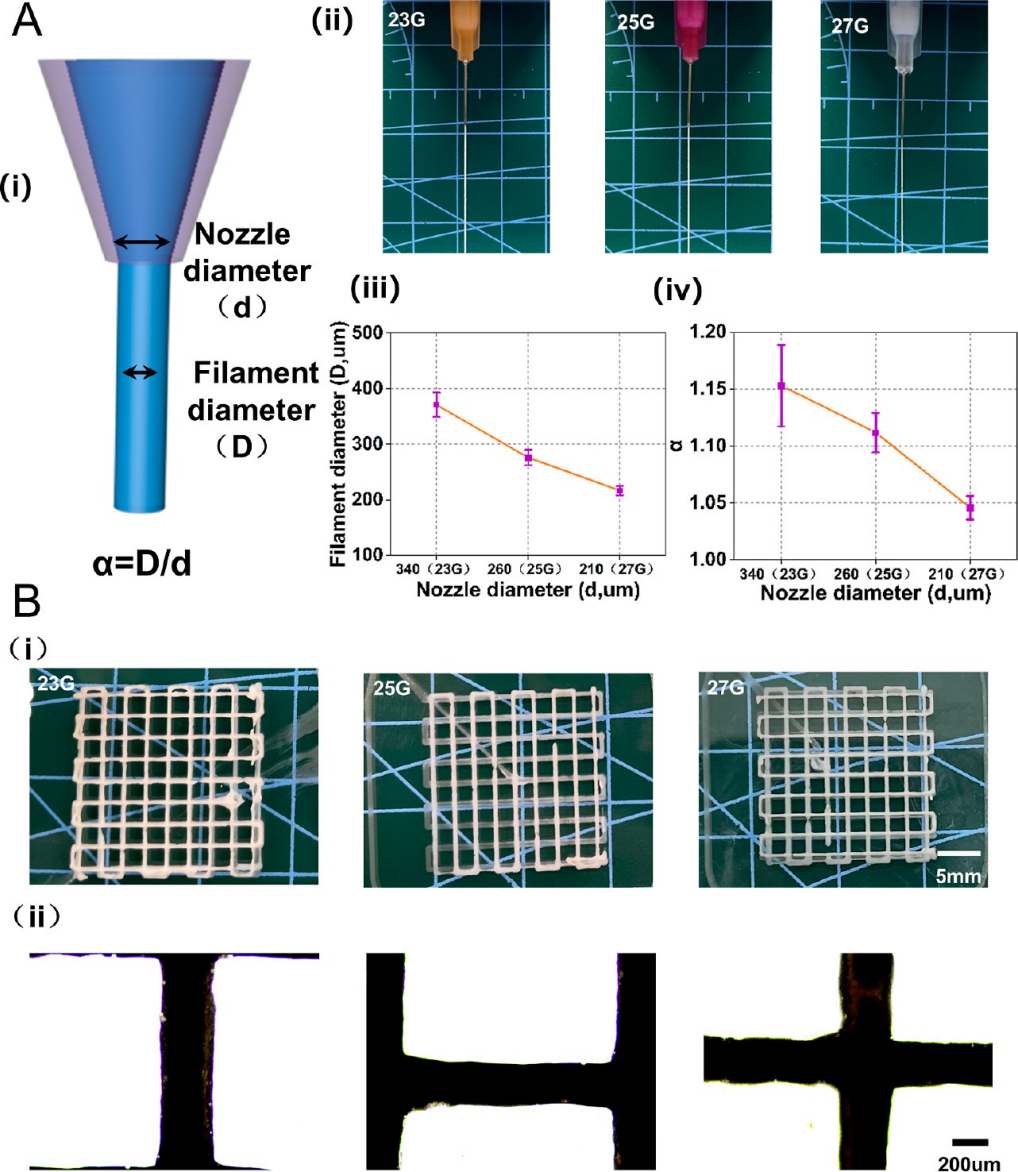
3D Printability of the hydrogel ink with regard to the stacking process. (A) Printability of ink with regard to the extrusion process. (i) Schematic illustration of the expansion phenomenon and definition, α = *D*/*d*. (ii) Effect of the nozzle diameter with 23, 25, and 27 and 27 G on expansion phenomenon. (iii) Extruded filament diameter and (iv) α. (B) Effect of printing accuracy. (i) Photographs of 3D printed lattice patterns using different nozzles with 23, 25, and 27 G. (ii) 25 G nozzle print rendering.

Figure 6 indicates that we achieve the optimal hydrogel ink, which allows for the continuous extrusion of a filament with a diameter nearly equal to the inner diameter of the conical nozzle. However, the filament still expands when it is extruded from the nozzle. During the extrusion process, the hydrogel ink expanded due to the release of shear stress, as shown in Figure 7A(i).

To investigate the 3D printability, we have characterized the expansion degree of the filament during extrusion while varying the nozzle diameter (23, 25, and 27 G). The results are presented in Figure 7A(ii). Here, we introduce α = *D*/*d* to describe the expansion degree of the filament, where *D* is the filament diameter, and *d* is the nozzle diameter. Figure 7A(iii,iv) shows that α decreases with the nozzle diameter. This may be because the ink underwent greater shear stress through a nozzle with a smaller diameter, thereby resulting in more strain. Using different nozzles, we printed samples with the same lattice pattern [Figure 7B(i)]. The optical images (optical microscope, Nikon) of the printed pattern are shown in Figure 7B(ii). It can be seen that the junction of the filaments is well fused, and the printing process is basically stable.

To validate the filament state, we have characterized the internal structures of printed architectures by SEM, which presents a continuous, interconnecting matrix between the ceramic flakes [Figure 6D(i–iii)]. According to our strategy, the LA rheology modifier doped into the macromolecular chain network serves two purposes: it provides an ideal extrusion state for the ink and helps the oxidizing agent that can be confined in the printed architecture to endow the architecture with the capability of polymerizing biomimetic dopamine. To further present the extrusion-flow behavior of the hydrogel ink with the LA rheology modifier doping, we have assessed the rheological characteristics of the hydrogel ink (see Section S3 and Figure S7 in the Supporting Information). First, as the content of LA increases, the modulus increases. As the *G*′ (storage modulus) and *G*″ (loss modulus) of the ink remain unchanged over time, DN–PDA–LA exhibits a steady state plateau when an equilibrium has been established, which is helpful to maintain a steady state of the printing process (Figure S7A). After that, we can observe that the hydrogel ink exhibits shear-thinning properties, as shown in Figure S7B. The viscosity of DN–PDA–LA inks decreases with increasing shear rate, indicating a shear thinning behavior of the hydrogel ink at room temperature. The shear thinning enhances the fluidity of the printing material, thereby making the ink extrude through the 3D printing nozzle. Hydrogel is a type of polymer material that possesses favorable viscoelastic and deformable properties. With regard to the viscoelasticity and rapidly thixotropic recovery, as depicted in Figure S7C,D, when the ink experiences mechanical deformation, its elastic and viscous responses are represented by its storage modulus (*G*′) and loss modulus (*G*″), respectively. The DN–PDA ink suitable for 3D printing possessed a high *G*′ to provide shape retention. Moreover, the hydrogel ink exhibits the ability to recover its previous state under low strain (1%) after being subjected to a flow state at high strain (400%), showcasing the rapid self-healing and remolding behaviors.

After the printed architecture was soaked into SN-DA solution [Figure 6D(iv–vi)], the disk-like clay flakes microstructure changed to a structure similar to the plicated byssal adhesive plaque. Distinctive microfibril bundles, ranging from 1 to 10 μm in diameter, were observed in the interpenetrating channel from the neighboring polymer matrix. In fact, the observed microfibril microstructure was also found in other PDA-based hydrogels,^18^ indicating that the hydrogels exhibited outstanding adhesive characteristics. The microfibrils were formed from complexation of the PDA and DN polymer matrix because dopamine can form the intermolecular interactions with a variety of polymers and additively generate unusual structures. As expected, the complexation of DN–PDA did not damage the 3D printed architectures because we confined the oxidation and cross-linking processes to the printed architectures.

**Figure 8:**
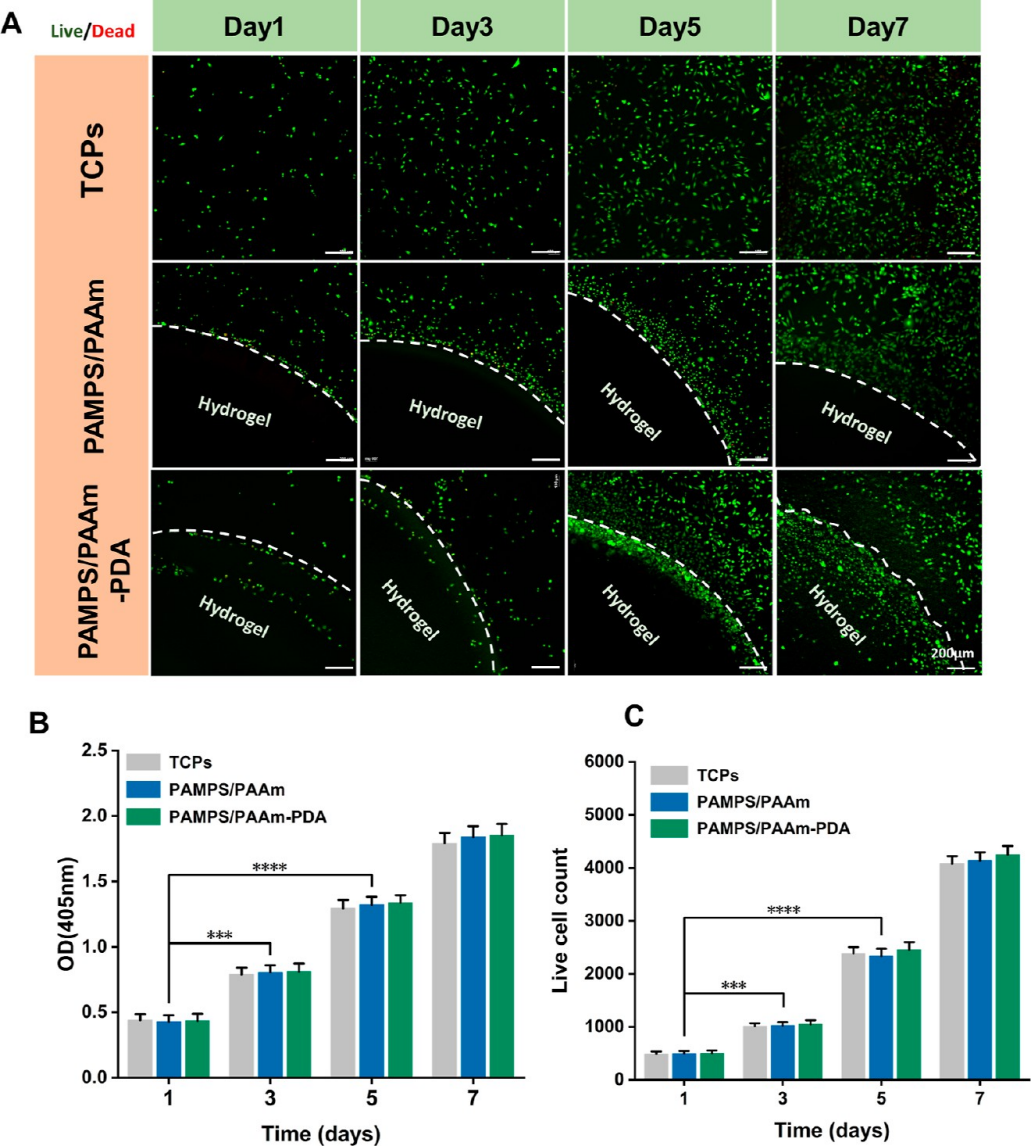
In vitro biocompatibility of the PAMPS/PAAm hydrogel and the PAMPS/PAAm-PDA hydrogel. The group without material culture was set as the blank control group (TCPs). (A) Live (green)/dead (red) fluorescence microscope images of L929 fibroblasts cultured with the hydrogels for 1, 3, 5, and 7 days, respectively. The white dash line represents the interface between the hydrogel and cells. (B) Viable cell count obtained from the live/dead staining assay showing the growth behaviors of cells on different hydrogels compared with the control panel. (C) CCK-8 results of the control, PAMPS/PAAm hydrogel, and PAMPS/PAAm-PDA hydrogel on 1, 3, 5, and 7 days. Sample size, *n* = 3, The values are shown as mean ± standard deviation (SD), ***P < 0.001, ****P < 0.0001, compared with the control group, TCPs.

Even though the biocompatibility of PAAm, alginate, Laponite, and PDA has been widely demonstrated, we still characterized the effect of biomimetic PDA on the biocompatibility of the DN(PAMPS/PAAm)-PDA hydrogel composites. In the cytotoxicity assay, we used L929 fibroblasts to culture with the PAMPS/PAAm-PDA hydrogels and PAMPS/PAAm hydrogels, respectively (Figure 8). The control group was set as cells without fibroblasts cultured (24-well tissue culture plates, TCPS). We evaluated the cell proliferation by the live/dead assay and the CCK-8 test. Fluorescent microscope images (fluorescence microscope, Nikon) were collected at different cultured times, that is, day 1, day 3, day 5, and day 7, respectively. As seen from Figure 8A, the microscope images showed that the vast majority of L929 fibroblasts remained viable throughout the 3 days of culture without any statistical differences between groups. The cells proliferated with increasing incubation times for all samples. After 3 and 5 days of culture, a large number of cells aggregated on the PAMPS/PAAm-PDA hydrogels and formed a cell colony. Over the period of coculture, the cells grew and proliferated well on the PAMPS/PAAm-PDA hydrogels. Consistent with these findings, the results of the CCK-8 assay revealed that the densities of L929 cells increased continuously over time without statistical difference between the three groups (Figure 8B,C).^20^ The results prove that the PAMPS/PAAm-PDA hydrogels have favorable biocompatibility. The active catechol groups on PDA facilitate cell affinity through conjunction with reactive groups (amino groups, carboxyl groups, and catechol groups) on cell membranes.^20^ Although the PAMPS/PAAm-PDA hydrogels have shown good biocompatibility and cell affinity in vitro, they warrant further evaluation for in vivo cell proliferation and differentiation capabilities.

## Conclusions

In summary, we demonstrate a tough, flexible, adhesive, and biocompatible PAMPS/PAAm-PDA hydrogel that can be customized into complex architectures such as lizard, ear, gecko, and fish by 3D printing. The 3D architectures exhibit superior adhesion to a variety of surfaces, including hydrophobic polythene and leaves as well as hydrophilic tissue, rock, metal, and wood. The reversible cross-linking of the PDA and PAMPS dissipates mechanical energy under deformation, and the long chain PAAm network as well as nanoceramic maintain high elasticity of the hydrogel. We have also established a theoretical framework to quantify the contribution of the interpenetrating networks to the overall toughness of hydrogels, which provides guidance for the rational design of hydrogels with the desired properties. The strategy of complexing mussel inspired PDA with PAMPS/PAAm results in repeatable adhesion and biocompatibility while combining toughness and water retention into a customized 3D interpenetrating network. We added a table in the Supporting Information, which lists the main hydrogel composition, cross-linker, initiator, curing condition, 3D printability, mechanical strength, and adhesion (Table S1) to compare our work with some published papers. Our work demonstrates a new method to print adhesive, tough, and biocompatible interpenetrating hydrogels, which promise broad potential applications, including but not limited to biomedical engineering, intelligent, soft robotics, and intelligent superabsorbent devices.

## Supporting Information Available

Materials processing and experimental procedures, microstructure characterization, rheology characteristics assay, mechanical behavior characterization, adhesion tests, in vitro biocompatibility and bioactivity, details of the network model, tensile mechanical behavior of the hydrogel, free-form DN(PAMPS/PAAm)-PDA 3D architecture, effect of SN-DA cross-linking time on the printed FN architectures, FN hydrogel samples, effect of soaking time on the FN samples, mechanical behavior of PAMPS/PAAm-PDA hydrogels, characterization results of the microstructures, rheological characteristics, and comparison of our work and literature (PDF)
Movie S1: The DN-PDA hydrogel showed a flexible feature when it was bent and released (MP4)
Movie S2: The 3D printed DN-PDA hydrogel was repeatedly bent and compressed to a large extent, and it recovered automatically and rapidly (MP4)
Movie S3: The 3D printed DN-PDA hydrogel adhered to the surface of polythene (Petri dish) (MP4)
