# Supplementary material for: 3D Printing of Interpenetrating Network Flexible Hydrogels with Enhancement of Adhesiveness
Source: ACS Appl Mater Interfaces. 2023 Aug 24;15(35):41892–905. doi: 10.1021/acsami.3c07816 (PMC10620755; doi:10.1021/acsami.3c07816)
Supplement: Supplementary file 1 — am3c07816_si_001.pdf [file am3c07816_si_001.pdf]

Supporting Information

3D Printing of Interpenetrating Network Flexible Hydrogels with Enhancement of Adhesiveness

Lei Zhang[1,2,+], Huifeng Du[3,+], Xin Sun[4], Feng Cheng[5], Wenhan Lee[4], Jiahe Li [4,6], Guohao Dai[4,&], Nicholas Xuanlai Fang[3,*], and Yongmin Liu[1,5,#]

[1] *Department of Mechanical & Industrial Engineering, Northeastern University, Boston, Massachusetts 02115, United States*

[2] *State Key Laboratory of Primate Biomedical Research, Institute of Primate Translational Medicine, Kunming University of Science and Technology, Kunming, Yun Nan 650000, China*

[3] *Department of Mechanical Engineering, Massachusetts Institute of Technology, Cambridge, Massachusetts 02139, United States*

[4] *Department of Bioengineering, Northeastern University, Boston, Massachusetts 02115, United States*

[5] *Department of Electrical and Computer Engineering, Northeastern University, Boston, Massachusetts 02115, United States*

[6] *Department of Biomedical Engineering, College of Engineering and School of Medicine, University of Michigan, Ann Arbor, Michigan 48109, United States*

[+] *L.Z. and H. D. contributed equally to this work.*

[&] *Corresponding author. E-mail address: g.dai@northeastern.edu*

[*] *Corresponding author. E-mail address: nicfang@mit.edu*

[#] *Corresponding author. E-mail address: y.liu@northeastern.edu*

Section S1. Materials processing and experimental procedures

Acrylamide (AAM, purity ≥ 99.5%), 2-Acrylamido-2-methylpropanesulfonic acid (AMPS, purity ≥ 99.0%), N,N′-Methylenebisacrylamide (MBAA, purity ≥ 99.5%), ammonium persulfate (APS, purity ≥ 98.0%), sodium Alginate, N,N,N′,N′-Tetramethylethylenediamine (TEMED, purity ≥ 99.5%) and dopamine hydrochloride (DA) were purchased from Sigma-Aldrich Co., Ltd. Firstly, 2.5 g AAM was dissolved in 10 ml deionized water under stirring at room temperature. Subsequently, we added MBAA and APS in quantities of 0.012 and 0.1 times

the weight of AAM. All the chemical components were stirred by magnetic stirring apparatus for 10 mins to form the first network (FN) solution. The rheology modifier was made by a combination of laponite ceramic and 1 wt.% alginate solution, abbreviated as L-A, in which laponite was added in quantities of 0.020 times the weight of FN. L-A was slowly added into FN solution while kept stirring for 20 mins to form the FN ink.

All the printed architectures were fabricated using a 3D-bioprinting system (BioX, Cellink, Sweden) via room-temperature pneumatic syringe extrusion. The FN ink was injected into 3 cc sterile plastic cartridges (Cellink, Sweden) and loaded onto the 3D-bioprinting. We mainly used sterile high precision conical plastic nozzles (Cellink, Sweden) with 18 G and 22 G diameter in our experiments to print the samples. The required extrusion pressure depended upon the ink viscosity, nozzle diameter and printing speed. The extrusion pressure was fairly low throughout this work, ranging from 10-80 psi at the extrusion speed of 10-75 mm/s. The samples were directly printed onto glass petri-dish with the same tip's height and the nozzle's inner diameter to ensure an appropriate attachment to the substrates and between printed layers. Once printed, the architectures were directly immersed into the second network-dopamine solution (SN-DA), which comprised 2.276 g AMPS, 2.5 g AAM, 71 mg MBAA, 200 mg APS, 75 mg DA, 20 μl TEMED and 10 ml deionized (DI) water. To explore the effect of composition on the mechanical property, we fixed the above composition as FN-SN-DA-LA and adjusted the composition on this basis. It should be noted that in the entire experimental procedure, we did not apply extra curing techniques such as UV irradiation and temperature change.

Section 2. Microstructure characterization

Samples without cells were prepared for scanning electron microscopy (SEM, Hitachi S-4800, Japan) and energy-dispersive x-ray spectroscopy (EDS, Hitachi S-4800, Japan) by coating with 6 nm platinum metal via plasma sputtering prior to imaging.

Section 3. Rheology characteristics assay

The evolution of viscosity、storage modulus (G') and loss modulus (G") was measured using a rheometer (HR10, TA instrument, DE New Burg, USA). Gelation kinetics were studied using a cone-plate geometry of 0.9711°, a plate spacing of 22uM, and a diameter of 40mm. The complex viscosity of the ink was measured under a strain of 1.0% and a speed of 10rad/s by time scanning at the room temperature. The shear-thinning behavior of the ink was measured using a flow sweep at a shearing rate of 1.0-100.0 l/s. A strain sweep from 0.01% to 10000% strain at 10 Hz was conducted with a conditioning time of 3 seconds and a sampling time of 3 seconds to determine the maximum deformability of the ink under the given conditions. The viscosity and rapid self-healing and remodeling behavior of the hydrogel were evaluated using a cyclic strain test method, which was repeated five times. The test parameters for the low and high strain (cyclic strain) time scans were 1.0% strain, 10 Hz (low strain) and 400% strain, 10 Hz (high strain), respectively.

Section 4. Mechanical behavior characterization

Compression test was carried out at an ambient temperature of 25 °C with a constant strain rate of 1 mm/sec on Instron mechanical tester (5966U1706, USA). 10 mm × 10 mm × 10 mm (length × width × height) cubic samples were adopted to conduct the engineering stress-strain

behavior at a compressive strain of 85% with a constant displacement rate of 1 mm/s. In order to test the shape recovery ability, the samples were loaded to 85% strain for 100 continuous cycles, and the evolution of stress-strain curves over cycles was recorded. All the samples were wiped with tissue paper to remove the residual water from the surface prior to compression test. Mechanical tensile tests of the rectangle hydrogel sample (15 mm in width, 1 mm in thickness, and 25 mm in length) at room temperature were performed using an Instron mechanical tester (5966U1706, USA) at the stretching rate of 10 mm min^{-1}. The number of test samples is 3.

Section 5. Adhesion tests

The adhesive strength of the DN-PDA hydrogels was measured by uniaxial tensile tests. Various substrates, including porcine skin, polyethylene film, glass slide, and aluminum plate, were employed to investigate the adhesive properties through tensile-adhesion testing. The hydrogels with a bonded area of 20 mm in width, 2 mm in thickness, and 20 mm in length were applied to the surface of the specimens. The samples were pulled at a tensile speed of 5 mm/min using the mechanical testing machine (5966U1706, USA) under ambient conditions until their separation. The number of test samples is 3.

Section 6. In vitro biocompatibility and bioactivity

L929 fibroblasts, which are usually used as cell biocompatibility assay, were obtained from North Na Biotechnology Co. LTD, Yunnan, China. Live/Dead staining assay was used to evaluate the viability of cells cultured with hydrogels (a diameter of 2mm and a thickness of 1mm). At the temperature of 37 °C, L929 fibroblasts (1×10^4 mL^{-1}) were cultured with the hydrogel in the 24-

well plates (0.4 μm pores, Corning, USA) with cell culture media (MEM, Minimum Essential Medium) containing 10% fetal bovine serum, 1% penicillin and streptomycin in humid air containing CO_2 (5%). On day 1, 3, 5 and 7, the cells were incubated with 250 μL Calcein AM/PI detection working solution (Beyotime, China) for 30 minutes and subsequently observed under a fluorescent microscope. The proliferation of the cells cultured with hydrogel was measured using Cell Count Kit-8 (CCK-8, Beyotime, China). Specifically, L929 fibroblasts (0.3×10^4 mL^{-1}) were cultured with the hydrogels of the 96-well plates (0.4 μm pores, Corning, USA). On day 1, 3, 5 and 7, the well plates were filled with 10 μL CCK-8 solution. After incubation at 37 °C for an hour, the absorbance value was measured at 450 nm via a FlexStation 3 microplate reader (Molecular Devices, Japan). The number of test samples is 3.

Section 7. Details of the network model

The network alteration functions are expressed as following[1-2]:

$$g(n_{i0}, N_{i0}, \Lambda_i^{\max}) = \frac{N_i(N_{i0}, \Lambda_i^{\max})\sqrt{n_i(n_{i0}, \Lambda_i^{\max})}}{\sqrt{3}} = N_{i0}\sqrt{\frac{n_{i0}}{3}}\exp\left[(0.5q - p)\left(\Lambda_i^{\max} - v_i^{1/3}\right)\right]$$

$$g_0(n_{i0}, N_{i0}) = N_{i0}\sqrt{\frac{n_{i0}}{3}}$$

$$(p = q = 0.35)$$

$g'(I_{\max}, I_1)$ also defines the chain alteration[1-3]:

$$g'(I_{\max}, I_1) = \begin{cases} \exp\left[\alpha_1\left(\sqrt{I_{\max}/3} - 1\right)\right], \text{loading} \\ \exp\left[\alpha_1\left(\sqrt{I_{\max}/3} - 1\right)\right]\exp\left[-\alpha_2\left(\sqrt{I_{\max}/3} - \sqrt{I_1/3}\right)\right], \text{unloading}(i \geq m_c) \\ \exp\left[-\alpha_3\left(\sqrt{I_{\max}/3}\right) - \sqrt{I_1/3}\right], \text{unloading}(i < m_c) \end{cases}$$

where $\alpha_1 = 0.5, \alpha_2 = 4.0, \alpha_3 = 5.0$, and m_c is the maximum critical chain length. The probability distribution can be expressed as[4]: $B = -G - \frac{\kappa}{\sqrt{\pi}}\left[\sqrt{6n_i}e^{-G} + 3\bar{r}\sqrt{\pi n_i}\mathrm{erf}\left(\sqrt{G}\right) - \sqrt{6}e^{-Gn_i} - 3\bar{r}\sqrt{\pi}\mathrm{erf}\left(\sqrt{Gn_i}\right)\right]$, with $G = \frac{3\bar{r}^2}{2n_i}$ and $\bar{r} = \frac{\sqrt{3}}{2}\left(2\frac{V_0}{v_N}\right)^{\frac{1}{3}}/b$. Here b is the Kuhn segment length[4], and κ is the proportion of active sites in total area[3].

Section 8. Tensile mechanical behavior of the hydrogel

In addition to the uniaxial compression assay, we have also conducted the uniaxial tensile assay. The tensile stress-strain curves, including elastic modulus and toughness[5-7] of the FN-SN-DA-LA hydrogel, FN-0.5SN-DA-LA hydrogel, FN-0.5SN-2DA-LA hydrogel and FN-SN-DA-2LA hydrogel, are shown in **Fig. S5**. Among the hydrogel samples tested, FN-SN-DA-LA exhibits the highest toughness, reaching 273.97 KJ m^{-3}. Additionally, the fracture stress, the tensile strain and the tensile modulus are 107.59 KPa, 432%, and 87.18 KPa, respectively. In contrast, when we double the content of LA, the FN-SN-DA-2LA hydrogel exhibits the highest tensile stress and the elastic modulus, but the lowest tensile strain. Apparently, the deformation characteristic in tension is consistent with the deformation characteristic in compression. The balance of the interpenetrating network formation and polymerization of PDA has resulted in the balanced performance on mechanical characteristics and 3D printing.

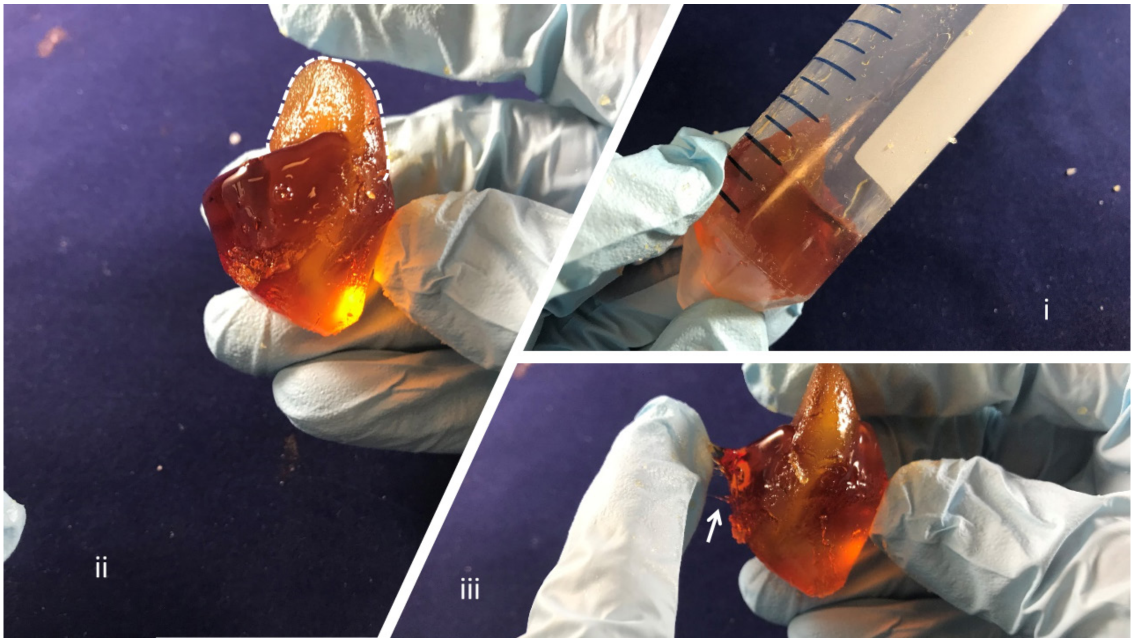

Fig. S1. Free-form DN(PAMPS/PAAm)-PDA 3D architecture. The printed 3D FN architecture (human ear) was put into a 50 ml conical bottom polypropylene tube. SN-DA solvent was added into the tube with the 3D printed sample, while the SN-DA liquid level did not exceed the sample. After 2-5 hours of cross-linking, the color of hydrogel was turned brown, indicating that dopamine polymerized. (i) and (ii) show the photographs of the sample before and after we took it out from the polypropylene tube. The printed architecture was cross-linked with the residual DN-PDA matrix, which gave rise to a conical shape same as the container. Because the added DA was too low to significantly consume the APS oxidizing agent, the FN in the architecture cross-linked a large amount of SN in the immersing solvent, before the SN-DA infiltrated into the printed architecture. The upper part of the DN(PAMPS/PAAm)-PDA architecture still maintained the original shape (outlined by the white dash line) because the upper part was immersed into the SN-DA solvent. The hydrogel finally integrated tough performance and adhesiveness (iii). Sticky extending fibers were observed, as pointed by the white arrow in (iii), when the hydrogel touched another surface.

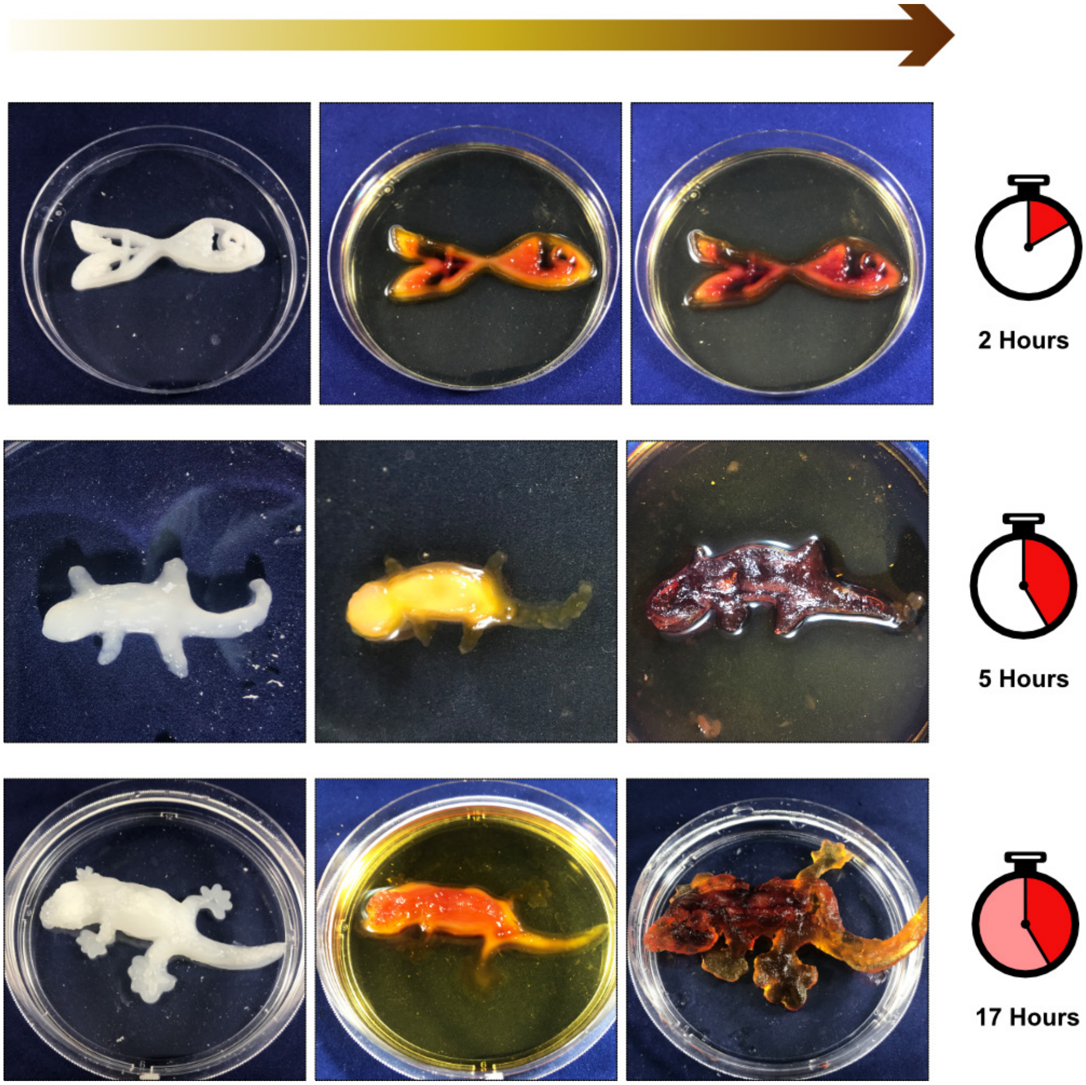

Fig. S2. Effect of SN-DA cross-linking time on the printed FN architectures. Photograph series of printed FN architecture changing to DN (PAMPS/PAAm)-PDA architectures (from left to right) with different cross-linking time (*e.g.*, 2, 5 and 17 hours). Biosynthesis of melanin can be an indicator of PDA polymerization. With the biosynthesis of melanin, dopamine was oxidized to DN-PDA polymer matrix. When the color of printed hydrogel was fully changed, adhesiveness and reversible mechanical features were obtained. In this case, the continuous long cross-linking time would give rise to the local swelling.

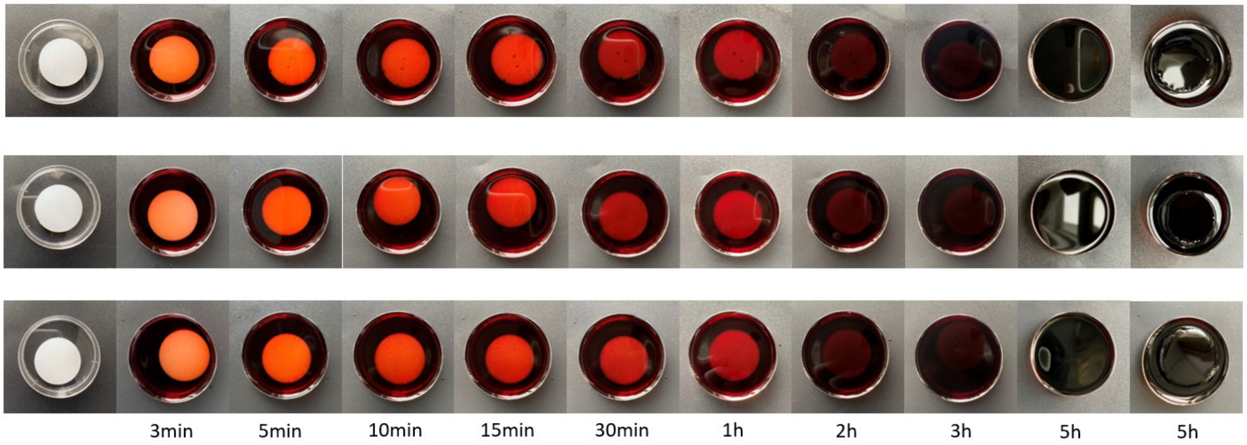

Fig. S3. FN hydrogel samples with diameter of 34 mm and thickness of 9 mm immersed in the second network solvent (SN-DA) to show the stability of DN-PDA hydrogel with different immersion times (n=3).

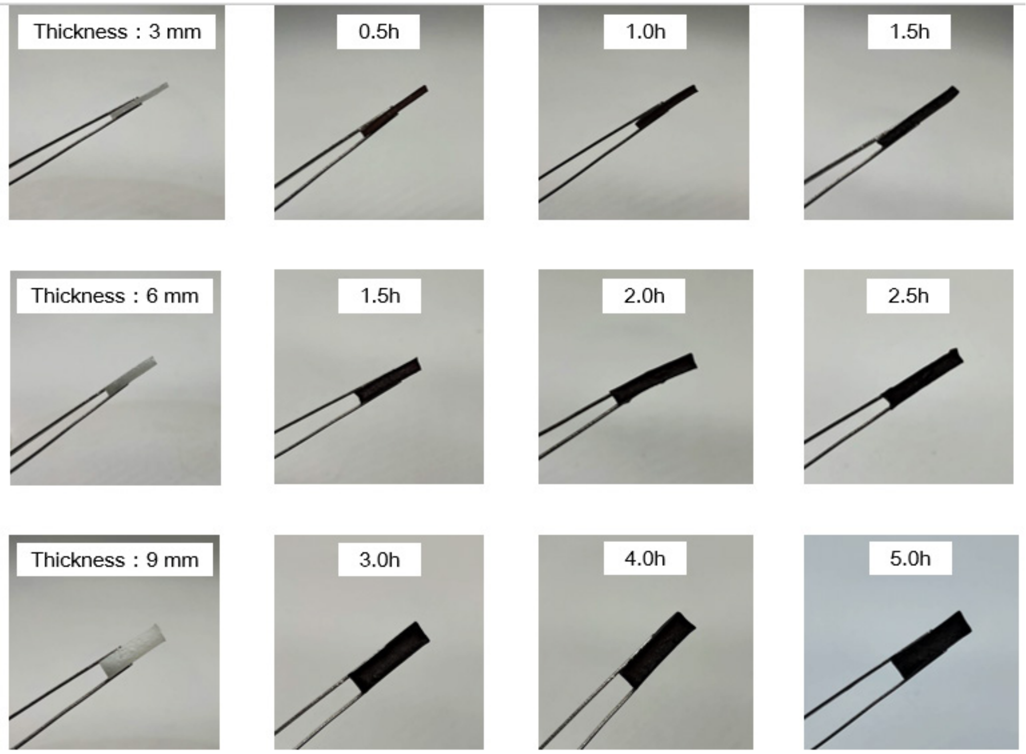

Fig. S4. Effect of soaking times on the FN samples with a diameter of 34 mm and thickness of 3, 6 and 9 mm respectively. The samples were cut into halves to observe the cross-linking and the diffusion extent inside of the samples after they were took out from the solvent. The photos show the cross section of the samples.

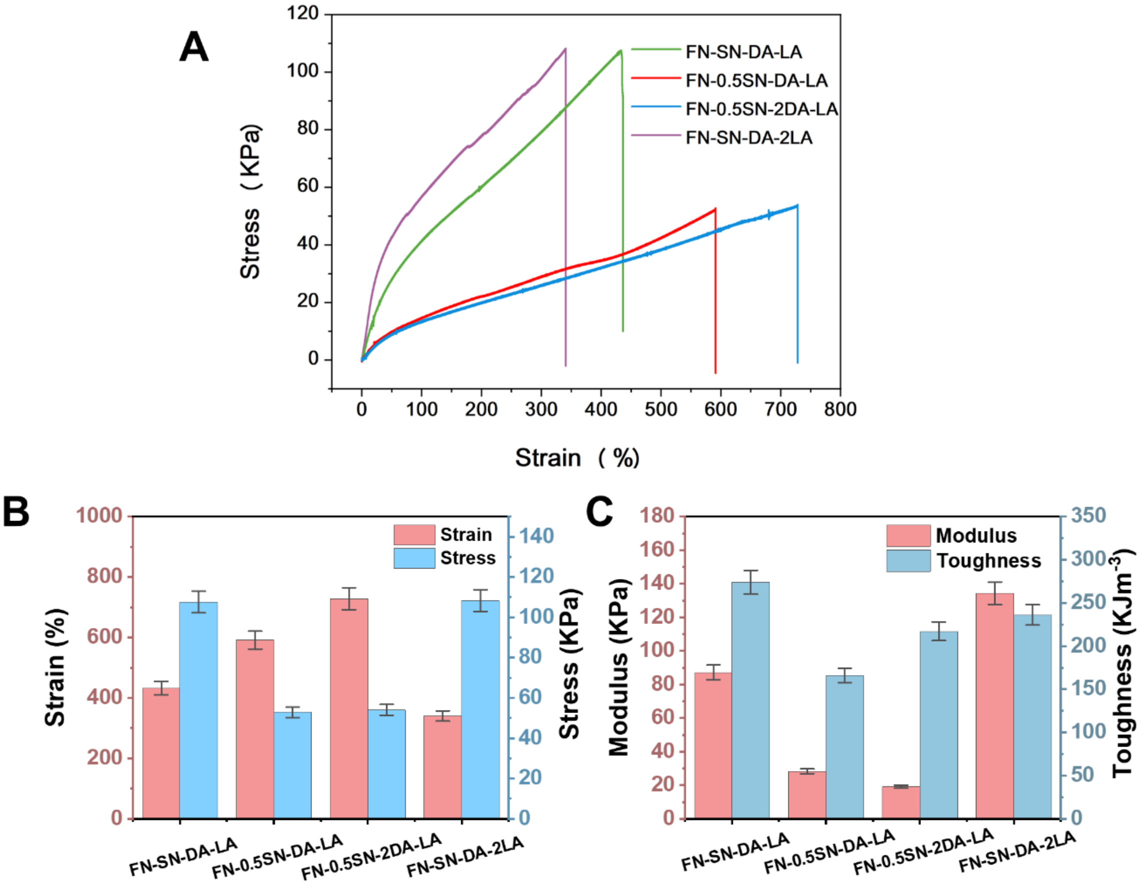

Fig. S5. Tensile of mechanical behavior of PAMPS/PAAm-PDA hydrogels. (A) Nominal tensile stress-strain curves of the FN-SN-DA-LA hydrogel, FN-0.5SN-DA-LA hydrogel, FN-0.5SN-2DA-LA hydrogel and FN-SN-DA-2LA hydrogel. (B-C) Tensile strain, tensile stress, elastic modulus and toughness deduced from the nominal tensile stress-strain curves of FN-SN-DA-LA hydrogel, FN-0.5SN-DA-LA hydrogel, FN-0.5SN-2DA-LA hydrogel and FN-SN-DA-2LA hydrogel.

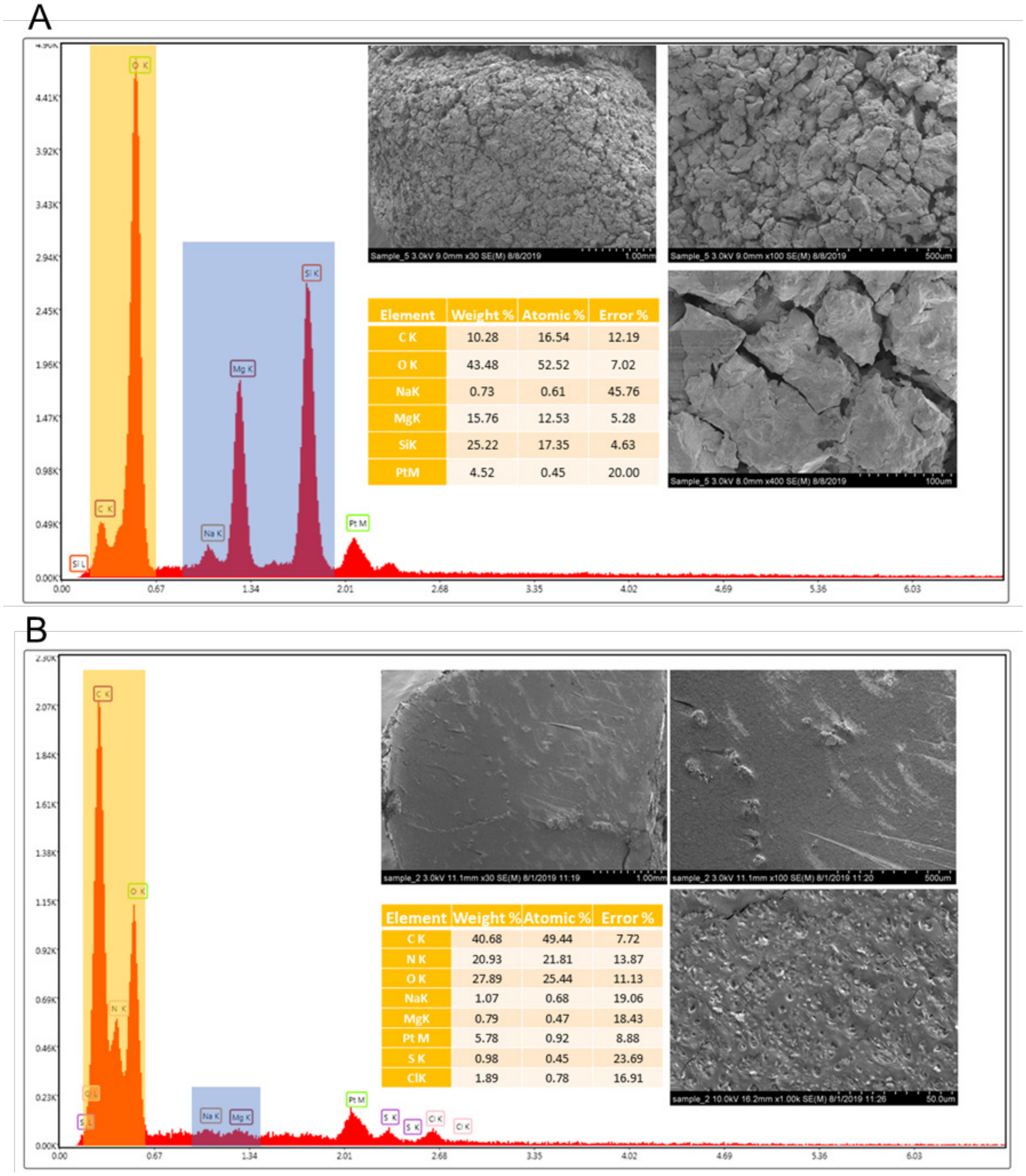

Element	Weight %	Atomic %	Error %
C K	10.28	16.54	12.19
O K	43.48	52.52	7.02
NaK	0.73	0.61	45.76
MgK	15.76	12.53	5.28
SiK	25.22	17.35	4.63
PtM	4.52	0.45	20.00

Element	Weight %	Atomic %	Error %
C K	40.68	49.44	7.72
N K	20.93	21.81	13.87
O K	27.89	25.44	11.13
NaK	1.07	0.68	19.06
MgK	0.79	0.47	18.43
Pt M	5.78	0.92	8.88
S K	0.98	0.45	23.69
ClK	1.89	0.78	16.91

Fig. S6. Characterization results of the microstructures of the hydrogels by scanning electron microscopy (SEM) and energy-dispersive X-ray spectroscopy (EDS). (A) EDS of the FN-SN-DA-2LA hydrogel. The inset is SEM image of the FN-SN-DA-2LA hydrogel. (B) EDS of the FN-0.5SN-2DA-LA hydrogel. The inset is SEM image of FN-SN-DA-2LA hydrogel. The tables are the element content of each hydrogel.

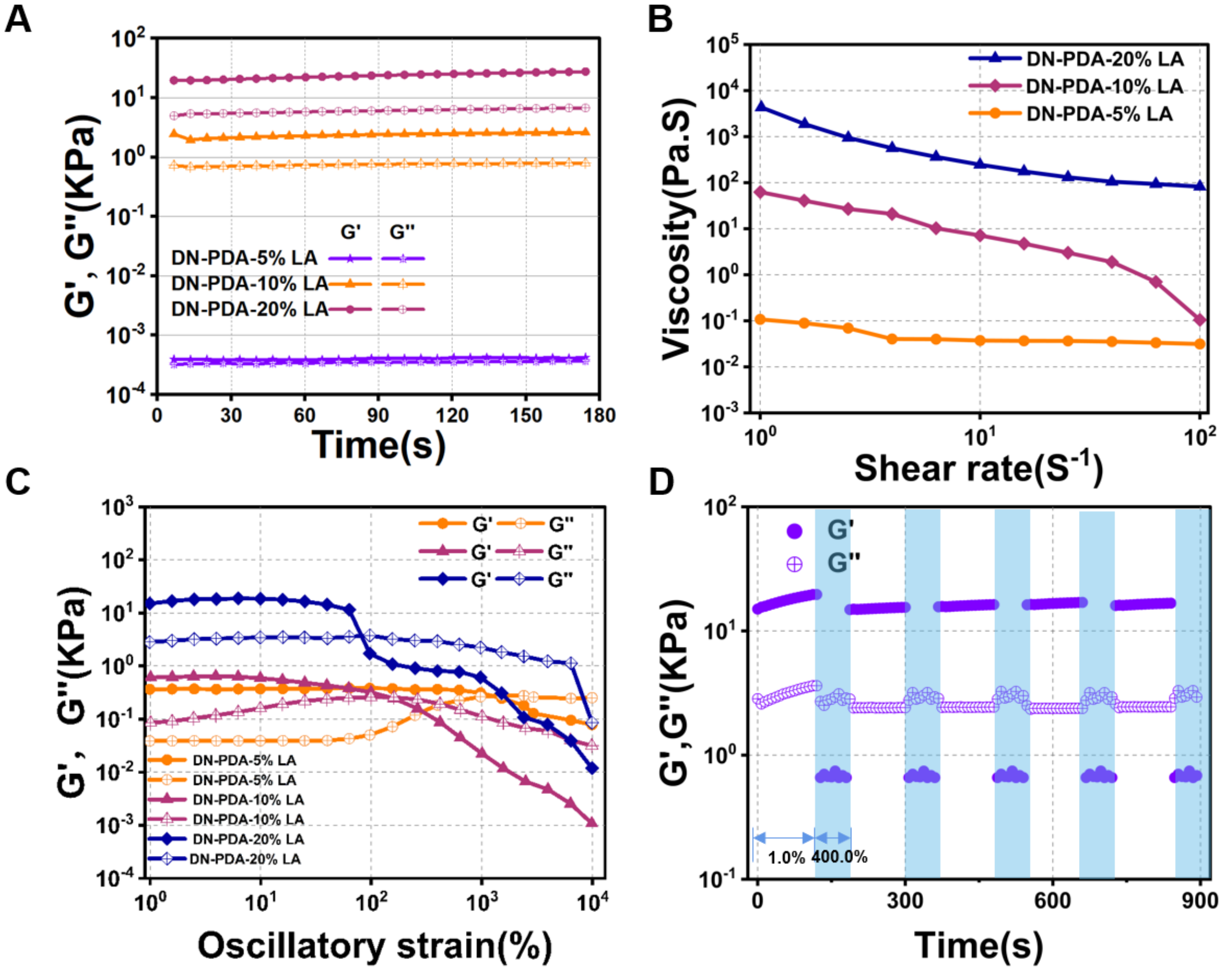

Fig. S7. Rheological characteristics of DN-PDA-LA inks: (A) storage modulus (G’) and loss modulus (G”) as a function of shear time; (B) viscosity as a function of shear rate; (C) storage modulus (G’) and loss modulus (G”) as a function of oscillatory strain; (D) rheological experiment of cyclic strain time scanning for the DN-PDA-20%LA hydrogel ink.

Table S1: Comparison of our work and literature.

	Hydrogel composition	Cross-linker	initiator	Curing condition	3D printing	Mechanical strength (Max)	Adhesion
Our work	Acrylamide, N,N′-Methylenebisacrylamide, Ammonium persulfate, Sodium alginate, Laponite, Dopamine hydrochlorid	MBAA，Ca^{2+}	APS, TEMED	Room temperature	Yes	5.8 MPa	Yes
Ref. 8	Carboxymethyl xylan, Fe3+ chloride hexahydrate,Acrylamide	MBAA	Potassium persulfate, Iron (III) chloride hexahydrate	Room temperature	-	-	-
Ref. 9	Acrylamide, 2-Acrylamido-2-methyl-1-propanesulfonic acid, Dopamine methacrylamide, 2,2-Dimethoxy-2-phenylacetophenone	MBAA	2,2-Dimethoxy-2-phenylacetophenone	UV	-	6.21 MPa	-
Ref. 10	Catechol-modified methacryloyl chitosan, catechol, FeCl3	$FeCl_3$	Lithium phenyl-2,4,6-trimethylbenzoylphosphinate, Fe3+	UV,	-	0.697 MPa	Yes
Ref. 11	Catechol sodium alginate, Acrylamide, N,N′-Methylenebisacrylamide, APS, Anhydrous $FeCl_3$	$FeCl_3$ MBAA	APS	60 °C 1 h	-	-	-
Ref. 12	Catechol-conjugated chitosan, Vanadium oxide sulfate hydrate	MBAA	Lithium phenyl-2,4,6-trimethylbenzoylphosphinate	UV	Yes	250 KPa	-
Ref. 13	Acrylamide, Four-armed PEG, APS	MBAA	APS	UV	Yes	1.6 MPa	-
Ref. 14	Bacterial cellulose, Acrylic acid, Polyethylene glycol diacrylate, 2,4,6-Trimethylbenzoyl-diphenylphosphine oxide, Sodium dodecyl sulfate	Polyethylene glycol diacrylate	2,4,6-Trimethylbenzoyl-diphenylphosphine oxide	90°C, 40 min Or, UV,	-	1.6 MPa	-
Ref. 15	Dopamine-carboxylated cellulose nanocrystal, Acrylamide, N,N′-Methylene bis-acrylamide, Potassium persulfate	MBAA	Potassium persulfate	65°C, 3h	-	81 KPa	Yes
Ref. 16	Polydopamine-reduced and sulfonated graphene oxide –poly(3,4-ethylenedioxythiophene), Acrylamide, APS, MBAA	MBAA	APS	Room Temperature	-	14 KPa	Yes
Ref. 17	PEGDA, Alginate, $CaCl_2$	Ca^{2+}	Lithium phenyl-2,4,6-trimethylbenzoylphosphinate,	Room temperature	Yes	200 KPa	-

References

1. Chagnon, G.; Verron, E.; Marckmann, G.; Gornet, L., Development of new constitutive equations for the Mullins effect in rubber using the network alteration theory. *International Journal of Solids & Structures* **2006,** *43* (22-23), 6817-6831.

2. Zhao, X., A theory for large deformation and damage of interpenetrating polymer networks. *Journal of the Mechanics & Physics of Solids* **2012,** *60* (2), 319-332.

3. Wang, Q.; Gao, Z., A constitutive model of nanocomposite hydrogels with nanoparticle crosslinkers. *J. Mech. Phys. Solids* **2016,** *94* (sep.), 127-147.

4. Dargazany, R.; Itskov, M., A network evolution model for the anisotropic Mullins effect in carbon black filled rubbers. *Int. J. Solids Struct.* **2009,** *46* (16), 2967-2977.

5. Al‐Ketan, O.; Rezgui, R.; Rowshan, R.; Du, H.; Fang, N. X.; Abu Al‐Rub, R. K., Microarchitected stretching‐dominated mechanical metamaterials with minimal surface topologies. *Adv. Eng. Mater.* **2018,** *20* (9), 1800029.

6. Avalle, M.; Belingardi, G.; Montanini, R., Characterization of polymeric structural foams under compressive impact loading by means of energy-absorption diagram. *Int. J. Impact Eng.* **2001,** *25* (5), 455-472.

7. Bai, R.; Yang, J.; Morelle, X. P.; Yang, C.; Suo, Z., Fatigue fracture of self-recovery hydrogels. *ACS Macro Lett.* **2018,** *7* (3), 312-317.

8. Li, N.; Sun, D.; Su, Z.; Hao, X.; Li, M.; Ren, J.; Peng, F., Rapid fabrication of xylan-based hydrogel by graft polymerization via a dynamic lignin-Fe3+ plant catechol system. Carbohydr. Polym. **2021**, 269, 118306.

9. Liu Y, Lee BP. Recovery property of double-network hydrogel containing mussel-inspired adhesive moiety and nano-silicate. J Mater Chem B. **2016** Oct 28;4(40):6534-6540.

10. Wang, L., Zhang, X. H., Yang, K., Fu, Y. V., Xu, T. S., Li, S. L., Zhang, D. W., Wang, L.-N., Lee, C.-S., A Novel Double-Crosslinking-Double-Network Design for Injectable Hydrogels

with Enhanced Tissue Adhesion and Antibacterial Capability for Wound Treatment. Adv. Funct. Mater. **2020**, 30, 1904156

11. Zhou, H. R.; Huang, J.; Chen, M.; Li, Y.; Yuan, M.; Yang, H., Effect of metal ions with reducing properties on hydrogels containing catechol groups. Colloids Surf., A **2021**, 631, 127657.

12. Li, N.; Xiang, Z.; Rong, Y.; Zhu, L.; Huang, X., 3D Printing Tannic Acid-Based Gels via Digital Light Processing. Macromol. Biosci. **2022**, 22 (4), 2100455.

13. Sun, W.; Xue, B.; Li, Y.; Qin, M.; Wu, J.; Lu, K.; Wu, J.; Cao, Y.; Jiang, Q.; Wang, W., Polymer-Supramolecular Polymer Double-Network Hydrogel. Adv. Funct. Mater. **2016**, 26 (48), 9044-9052.

14. Roig-Sanchez, S.; Kam, D.; Malandain, N.; Sachyani-Keneth, E.; Shoseyov, O.; Magdassi, S.; Laromaine, A.; Roig, A., One-step double network hydrogels of photocurable monomers and bacterial cellulose fibers. Carbohydr. Polym. **2022**, 294, 119778.

15. M. J. Hossen, S. D. Sarkar, M. M. Uddin, C. K. Roy, M. S. Azam, ChemistrySelect **2020**, 5, 8906.

16. Gan, D., Huang, Z., Wang, X., Jiang, L., Wang, C., Zhu, M., Ren, F., Fang, L., Wang, K., Xie, C., Lu, X., Graphene Oxide-Templated Conductive and Redox-Active Nanosheets Incorporated Hydrogels for Adhesive Bioelectronics. Adv. Funct. Mater. **2020**, 30, 1907678

17. Hong, S.; Sycks, D.; Chan, H. F.; Lin, S.; Lopez, G. P.; Guilak, F.; Leong, K. W.; Zhao, X., 3D Printing: 3D Printing of Highly Stretchable and Tough Hydrogels into Complex, Cellularized Structures. Adv. Mater. **2015**, 27 (27), 4034-4034.
